# Microbiologically influenced corrosion (MIC) potential of bentonite microorganisms: implications for a deep geological repository for nuclear waste

**DOI:** 10.1007/s11274-026-05039-0

**Published:** 2026-06-01

**Authors:** Kateřina Černá, Saqlain Saqib Mukhtar, Richard Bureš, Gabriela Alfaro-Espinoza, Andrea Koerdt, Jakub Říha, Veronika Hlavackova, Jan Stoulil

**Affiliations:** 1 Institute for Nanomaterials, Advanced Technologies and Innovation, Technical University of Liberec, Studenstká 1402/2, Liberec, 46117 Czech Republic; 2 University of Chemistry and Technology, Technická 5, Prague, 166 28 Czech Republic; 3 Bundesanstalt für Materialforschung und -prüfung (BAM), Unter den Eichen 87, Berlin, 12205 Germany

**Keywords:** Microbiologically influenced corrosion (MIC), Deep geological repositories (DGR), Carbon steel canisters, Bentonite-associated microorganisms, Biofilm

## Abstract

The integrity of carbon steel canisters in deep geological repositories (DGRs) for high-level radioactive waste (HLW) may be compromised by microbiologically influenced corrosion (MIC) driven by bentonite-associated or naturally occurring subsurface microorganisms. This study investigates the MIC potential of anaerobic microbial consortia enriched from Czech bentonite Černý Vrch (BCV). It represents the first phase of a comprehensive research project on MIC in BCV bentonite, aimed at identifying the most corrosive environments and taxa, progressing toward mechanistic studies of microbial electron transfer and corrosion behavior under near-repository conditions. Carbon steel coupons were incubated in selective media inoculated with BCV targeting nitrate-reducing bacteria (NRB), sulfate-reducing bacteria (SRB), heterotrophs, acetogens, and methanogens, under static and dynamic flow conditions. A two-stage (2- and 3-month-long, respectively) batch experiment was performed, with the second stage employing inocula from the first to enrich MIC-active consortia. Corrosion rates were quantified, and microbial communities analyzed using qPCR and 16 S rRNA amplicon sequencing. Highest corrosion rates were observed in Nitrate Broth (targeting nitrate reducers, NRB), R2A (heterotrophs), and Postgate (sulphate reducers, SRB) media, reaching 60 μm·a⁻¹, 31 μm·a⁻¹, and 33 μm·a⁻¹, respectively. Corrosion localization was observed only in Nitrate Broth media (maximum penetration depth nearly 40 μm). Organic-rich media supported greater microbial diversity and activity. Dynamic flow conditions simulating worst-case scenarios significantly increased corrosion. In Nitrate Broth, sterile samples rose 11.8-fold (211 μm·a⁻¹) and biotic samples 6.5-fold (125 μm·a⁻¹) compared to static conditions. In R2A, sterile samples increased 11.5-fold (28 μm·a⁻¹) and biotic 9.7-fold (37 μm·a⁻¹). Clostridia, Bacilli, and SRBs were identified as key corrosion-inducing organisms in the studied systems. Our findings identify NRB as a potential corrosion threat, refine MIC risk assessments for DGRs, and improve predictions of canister longevity and repository safety.

**Supplementary Information:**

The online version contains supplementary material available at 10.1007/s11274-026-05039-0.

## Highlights

Bentonite-associated microbial consortia accelerated carbon steel corrosion under repository conditions.

Dynamic flow increased MIC rates in biotic samples up to 72-fold compared to static sterile systems.

Fermentative, acetogenic, and sulfate-reducing bacteria were key taxa linked to corrosion.

Biofilm-associated communities on metal surfaces differed from bulk cultures.

Findings improve MIC risk assessment for deep geological repositories.

**Supplementary Information:**

The online version contains supplementary material available at 10.1007/s11274-026-05039-0.

## Introduction

Nuclear power supports national energy transitions by delivering reliable baseload electricity with minimal greenhouse gas emissions (IAEA 2021). However, the long-term sustainability of nuclear energy depends on the safe management and disposal of high-level radioactive waste (HLW). Deep geological repositories (DGRs) are widely considered to be the safest long-term solution for high-level radioactive waste (HLW) disposal (Harper et al. 2024). In a typical DGR design, waste is sealed in a corrosion-resistant metal canister and placed hundreds of meters underground within a multi-barrier system that includes both engineered and natural barriers (IAEA 2009).

Highly compacted bentonite clay is a widely accepted component of engineered barrier system (EBS) in many national disposal concepts, including those under development in the Czech Republic (Havlová et al. 2020; Kumpulainen et al. 2022; OECD and Nuclear Energy Agency 2003). Bentonite is used as a buffer around waste canisters and as a backfill in disposal tunnels due to its favorable properties, including low hydraulic conductivity, self-sealing behavior, swelling and sorption capacity, and long-term physicochemical stability (Posiva And Oy 2017; SKB, 2010a, 2010b). These properties limit groundwater transport, retard radionuclide migration, and provide mechanical protection to the canister.

Despite its favorable characteristics, bentonite is not geochemically inert. Long-term exposure to elevated temperature or alkaline leachates from cementitious components can trigger illitization, reducing swelling capacity and sealing efficiency (Kaufhold et al. 2023; Ohazuruike And Lee 2023). Desiccation during early repository evolution can further lead to shrinkage and cracking prior to full resaturation (Lang et al. 2018; Noh et al. 2025; Svensson et al. 2011). Furthermore, bentonite contains a diverse community of indigenous microorganisms such as heterotrophic aerobes and anaerobes (Bartak et al. 2023; Engel et al. 2023), sulfate-reducing bacteria (SRB) (Bengtsson And Pedersen 2016; Masurat et al. 2010), fermenters (Vachon et al. 2021), acetogens (Bartak et al. 2024; Svensson et al. 2011), or iron-reducing bacteria (IRB) (Gilmour et al. 2021).

Microbial dormancy is expected under the initially harsh repository conditions; however, microbial reactivation following cooling or water ingress may initiate biogeochemical processes with implications for repository safety. Of particular concern is microbiologically influenced corrosion (MIC), which can significantly accelerate canister degradation under anoxic conditions (Rajala et al. 2019). Sulfate reducers (SRB) are of particular concern because they can produce corrosive sulfides, which pose a threat, especially in copper-based canisters (Harper et al. 2024; Ma et al. 2022). Iron-reducing bacteria (IRB) can reduce structural Fe³⁺ in smectites and promote mineralogical alterations under certain experimental conditions (Kim et al. 2004; Libert et al. 2011). These processes can drive redox-mediated mineral transformations, secondary iron mineral formation, silica mobilisation, and degradation of geomechanical properties in MX-80 bentonite under repository-relevant conditions (Gilmour et al. 2021, 2022). More broadly, heterotrophic degradation of organic matter, gas generation, pH shifts, and biofilm formation can further influence corrosion processes and bentonite performance (Marshall et al. 2015).

MIC is a multifactorial process involving several possible mechanisms that often occur simultaneously and interactively, as comprehensively reviewed by (Ahmed et al. 2024; Enning And Garrelfs 2014; Knisz et al. 2023; Liu et al. 2023; Taghavi Kalajahi et al. 2024). One important mechanism is chemical MIC (CMIC), driven by corrosive metabolic products. Under DGR conditions, sulfate-reducing bacteria (SRB) are major contributors to CMIC by producing hydrogen sulfide in the bentonite buffer (Bengtsson et al. 2015). The biogenic sulfide reacts with metal surfaces, forming metal sulfides (Černoušek et al. 2020; Hajj et al. 2010), and is particularly aggressive toward copper-based canisters under anaerobic conditions (Chen et al. 2011; King 2013). Consequently, extensive research programs in Finland, Sweden, and Canada have focused on sulfide-induced corrosion and its inclusion in canister design and safety assessments (Harper et al. 2024; Jang et al. 2025; King et al. 2024; Lilja 2010).

In carbon steel systems, reaction with biogenic sulfide typically leads to the formation of iron sulfide phases, most notably mackinawite (FeS), which can promote localized electrochemical activity and hydrogen evolution at the metal surface (Ning et al. 2013). Hydrogen sulfide produced by bacteria also lowers pH, and S^2-^ species in general inhibit hydrogen recombination, facilitating hydrogen uptake into steel and increasing susceptibility to sulfide stress cracking and hydrogen-assisted cracking, particularly at corrosion pits and elevated temperatures (Turnbull 2009). Additional corrosion products, including greigite, pyrite, magnetite, and siderite, may form under DGR-relevant conditions, with varying electrochemical properties that can influence localized corrosion behavior(Cornell And Schwertmann 2003; Ning et al. 2013; Telford et al. 1990).

In addition to CMIC, electrochemical MIC (EMIC) represents a distinct mechanism in which microorganisms directly accelerate anodic metal dissolution by directly withdrawing electrons from the metal surface (Enning And Garrelfs 2014). This process is limited to microorganisms capable of extracellular electron transfer, either directly or via redox-active mediators (Logan 2009) and has been identified among many microbial taxa, including sulfate-reducing bacteria, iron-oxidizing bacteria, or methanogens (An et al. 2020; Enning And Garrelfs 2014; Ueki et al. 2018). EMIC can generate distinct anodic and cathodic microenvironments that intensify pitting and crevice corrosion. The specific contribution of EMIC to corrosion in deep geological repositories (DGRs) remains unclear due to anoxic, low-nutrient conditions, long timescales, complex mineral–microbe interactions, and limited in situ data, making experimental modeling assessment challenging.

This knowledge gap is especially relevant for inland DGR concepts employing steel-based canisters in low-salinity groundwater environments, such as those in France, Switzerland, and the Czech Republic (King et al. 2024; Pospiskova et al. 2017). The Czech concept uses a stainless steel inner vessel encased in a thick carbon steel overpack for HLW (Hausmannová et al. 2023). MIC of carbon steel in bentonite environments – especially localized EMIC or galvanic effects on the outer carbon steel layer – remains less understood and is a subject of growing concern. Moreover, it is now evident that microbial risks are not limited to SRB alone. Recent studies indicate that microbial groups beyond SRB, including nitrate-reducing and iron-reducing bacteria, can substantially enhance localized carbon steel corrosion in bentonite systems (Shrestha et al. 2021). However, the relative roles and mechanisms of these microbial communities under repository-relevant conditions remain insufficiently understood.

The overall project integrates (i) systematic screening of the MIC potential of indigenous bentonite-associated microbial communities, (ii) targeted mechanistic investigations of corrosion processes and microbe–metal interactions, and (iii) corrosion simulations conducted under progressively more realistic repository conditions, including experiments in compacted bentonite. The results of each phase will be reported separately and together form a systematic framework for understanding MIC and supporting long-term safety assessments for radioactive waste disposal.

In this initial phase, we test the hypothesis that specific anaerobic microbial functional groups originating from bentonite, including heterotrophs, nitrate- and sulfate-reducing bacteria, acetogens, fermenters, and methanogens, can significantly contribute to carbon steel corrosion under controlled laboratory conditions. To address this, we compare corrosion behavior in static diffusion-limited systems and dynamic flow-through systems representing conservative, stress-test scenarios. The results provide a foundation for subsequent experimental phases and contribute to improved assessment of microbial risks in bentonite-based engineered barrier systems.

## Materials and methods

### Metal coupon preparation

Metal samples for all experiments were prepared from a cold-rolled steel sheet (EN 1.0330), containing C < 0.12%; Mn < 0.65%; *P* < 0.045%; S < 0.045%, by cutting it into 1 × 1 cm pieces. The sample surfaces were ground with P120 sandpaper. Each sample was then marked using an engraving machine and rinsed with distilled water, followed by absolute ethanol. Subsequently, the samples were weighed on an analytical balance (1601 MP8, Sartorius, Germany) prior to exposure. All samples were sterilized by dry heat at 170 °C for 2 h before the start of the experiment.

### Microbial inoculum and bentonite material

All experimental setups utilized Czech bentonite Černý Vrch (BCV), a magnesium–calcium-rich clay sourced and processed by Keramost Ltd., Czech Republic (Kašpar et al. 2021). This material served as the microbial inoculum across all experiments. Comprehensive mineralogical and geochemical characterization of BCV has been previously reported (Kašpar et al. 2021).

### Batch experiment design

We first conducted two subsequent batch experiments, further denoted as Batch experiments 1 and 2, to identify bentonite-indigenous microbial taxa capable of utilizing metal as a direct electron donor and potentially inducing localized microbially influenced corrosion (MIC) on carbon steel surfaces. This was assessed by comparing corrosion rates of biotic samples with sterile controls, with particular emphasis on the occurrence of corrosion localization features indicative of microbially mediated processes. The focus was on anaerobic microorganisms, particularly nitrate-reducing bacteria (NRB), anaerobic heterotrophs, sulfate-reducing bacteria (SRB), heterotrophic acetogens, and other fermentative microorganisms, and bentonite-associated methanogens, to evaluate their relevance to MIC processes in DGR.

To achieve this, various culture media (see below) were inoculated with 0.2 g of naturally moist bentonite per 20 mL of medium. This low concentration was intentionally chosen to use bentonite solely as a microbial inoculum while minimizing its potential chemical influence on corrosion processes. Each culture sample was supplemented with two carbon steel corrosion coupons (10 × 10 × 0.8 mm), and incubated under static conditions in the anaerobic chamber at ambient temperature (~ 26 °C) for two months (Experiment 1). The tested media included synthetic bentonite pore water (PW) and nitrate-enriched pore water (PWN), both of which have previously been linked to EMIC activity (Shrestha et al. 2021). The chemical composition is described in Table 1.

**Table 1:** Chemical composition of the culture media used in our experiment. PW - synthetic bentonite pore water, PWN - PW supplemented with nitrate, PGM - Postgate medium, LB - Lysogeny Broth, NRM - Nitrate Broth, R2A - R2A medium, ME - medium 120 (Koblitz et al. 2023). 100/25/0–100%/25%/0% of organic content compared to the original media prescription. Organic compounds where we varied their concentration are in grey. The composition of Trace element solution SL-10 and Wolin’s vitamin solution is in Supplementary Material 2, Supplementary Table S1

Compound (unit)	PW	PWN	PGM 100%	PGM 25%	PGM 0%	ME 100%	ME 25%	ME 0%	LB 100%	LB 25%	R2A 100%	R2A 25%	R2A 0%	NRM 100%	NRM 25%	NRM 0%
Ascorbic acid (mg)			1	1	1											
CaCl2 × 2 H2O (g)			0.1	0.1	0.1	0.25	0.25	0.25								
Ca2SO4 × 2 H2O (g)	0.0517	0.0517														
Casamino acids (g)											0.5	0.125	0			
Casitone (g)						2	0.5	0								
FeSO4 × 7 H2O (5% w/v) (mL)			10	10	10											
FeSO4 × 7 H2O (0.1% w/v) (mL)						2	2	2								
Glucose (g)											0.5	0.125	0			
KHCO3 (g)	0.044	0.044														
K2HPO4 (g)			0.5	0.5	0.5	0.35	0.35	0.35			0.3	0.3	0.3			
KH2PO4 (g)						0.23	0.23	0.23								
L-Cysteine HCl x H2O (g)						0.3	0.3	0.3								
Meat extract (g)														3	0.75	0
Methanol (50% v/v) (mL)						20	5	0								
MgSO4 × 7 H2O (g)			2	2	2	0.5	0.5	0.5			0.05	0.05	0.05			
NH4Cl (g)			1	1	1	0.5	0.5	0.5								
Na-DL-lactate (g)			2	0.5	0											
Na-acetate (g)						2.5	0.625	0								
Na-pyruvate (mg)											300	75	0			
Na-thioglycolate (mg)			1	1	1											
NaHCO3 (g)	0.546	0.546														
NaNO3 (g)	0.8163															
Na2S x 9 H2O (g)						0.3	0.3	0.3								
Na2SO4 (g)	0.0568	0.0568	1	1	1											
Na2SO4 × 7 H2O (g)	0.1725	0.1725														
NaCl (g)	0.0234	0.0234				2.25	2.25	2.25	10	10						
NaF (g)	0.0084	0.0084														
NaHCO3 (g)						2	2	2								
Peptone (g)														5	1.25	0
Potassium nitrate (g)														1	1	1
Proteose peptone (g)											0.5	0.125	0			
Sodium resazurin (0.1% w/v) (mL)			0.5	0.5	0.5	0.5	0.5	0.5								
Starch (g)											0.5	0.125	0			
Trace element solution SL-10 (mL)						1	1	1								
Tryptone (g)									10	2.5						
Wolin’s vitamin solution (10x) (mL)						1	1	1								
Yeast extract (g)			1	0.25	0	2	0.5	0	5	1.25	0.5	0.125	0			
**Distilled water (mL)**	**1000**	**1000**	**1000**	**1000**	**1000**	**1000**	**1000**	**1000**	**1000**	**1000**	**1000**	**1000**	**1000**	**1000**	**1000**	**1000**

Additional media were selected to enrich for specific functional groups: Postgate Medium (PGM) for sulphate-reducers (SRB), Lysogeny Broth (LB) for anaerobic heterotrophs and acetogens, Nitrate Broth Medium (NRM) for nitrate-reducers (NRB), R2A for slow-growing anaerobic heterotrophs, and Medium 120 (ME) (Koblitz et al. 2023) for methanogens targeting *Methanosarcina*, which was previously observed in BCV bentonite slurries (unpublished). Each medium type was tested in three formulations: full-strength (concentration of organic compounds exploitable as electron acceptors as in the original media formula, further denoted as 100% medium), reduced-organic (25% organic content compared to the original formula), and organic-free (0%). The compositions of all media are detailed in Table 1.

This gradient was designed to compare corrosion and microbial processes across nutrient-rich, reduced-organic, and nutrient-depleted conditions. Fully organic media served as compositionally complex standard controls, while zero-organic media most closely represent nutrient-depleted DGR environments; however, biomass production under these conditions was insufficient for reliable molecular analyses, particularly from corrosion coupon surfaces. Low-level organic supplementation (25%) was therefore introduced as a methodological tool to overcome biomass and detection limitations inherent to ultra-oligotrophic systems and to ensure sufficient microbial activity for reproducible analytical and molecular characterization. Organic limitation was expected to promote the use of alternative electron donors, including metallic iron, as a consequence of reduced availability of readily metabolizable organic substrates. Half of the samples were further amended with 0.2 g powdered metallic iron (mean grain size 120 μm; Carlo Erba, Italy) per 20 mL to promote the enrichment of MIC-active bacteria by providing an increased metal surface area within the experimental system. Duplicate samples were prepared for each biotic condition. Triplicate sterile controls (consisting of 100% medium without bentonite or iron powder) were included for each medium type to distinguish biotic corrosion from abiotic background processes. Bentonite was deliberately excluded from sterile controls, as its sterilization is technically challenging (Bartak et al. 2025) and could compromise the sterility of control samples. The sample overview is summarized in Table 2.

**Table 2:** List of samples in the Batch experiments 1 and 2. Each sample contained two carbon steel corrosion coupons. PW - synthetic bentonite pore water, PWN - PW supplemented with nitrate, PGM - Postgate medium, LB - Lysogeny Broth, NRM - Nitrate Broth, R2A - R2A medium, ME - medium 120 (Koblitz et al. 2023). 100/25/0–100%/25%/0% of organic content compared to the original media prescription

	Batch experiment 1			Batch experiment 2	
Sample composition	BCV innoculated, with powder iron (+ *P*)	BCV iInnoculated, without powder iron	Sterile, without powder iron	BCV innoculated, with powder iron (+ *P*)	BCV innoculated, without powder iron
PW	2	2	3	2	2
PWN	2	2	3	2	2
PGM100	2	2	3	2	2
PGM25	2	2		2	2
PGM0	2	2		2	2
LB100	2	2	3	2	2
LB25	2	2		2	2
R2A100	2	2	3	2	2
R2A25	2	2		2	2
R2A0	2	2		2	2
NRM100	2	2	3	2	2
NRM25	2	2		2	2
NRM0	2	2		2	2
ME100	2	2	3	2	2
ME25	2	2		2	2
ME0	2	2		2	2
**Sum samples**	**32**	**32**	**21**	**32**	**32**

At the end of Experiment 1, 1 mL of culture medium was taken from each inoculated sample and transferred into fresh media (same composition) containing new carbon steel coupons to initiate a second batch (Experiment 2). These follow-up samples were incubated for approximately three months. Because only culture medium (with only traces of bentonite) was transferred, Experiment 2 served to assess the activity of microbial populations that had successfully proliferated under the conditions of Experiment 1, rather than the full bentonite community.

After exposure, the corrosion coupons were removed from the culture media. In samples with 25% and 100% organic content, one coupon per culture sample was swabbed to assess biofilm composition (hereafter referred to as swab), then dried under anoxic conditions for subsequent mass loss and microscopic analyses (see below). Coupon swabs from the 0% organic cultures were not genetically analysed due to insufficient biomass for reliable genetic analysis (DNA concentrations below detection limits in the bulk sample and few tested swab samples). This limitation directly constrained molecular characterization in these systems and represents a methodological trade-off between representativeness of ultra-oligotrophic conditions and analytical feasibility, which motivated the inclusion of low-level organic amendments in other treatments. The remaining coupons from all treatments were left intact and dried anoxically for later mass loss measurements and microscopic examination. A 5 mL liquid per culture sample was further collected for microbial composition analysis (hereafter referred to as bulk medium), centrifuged at 11,000 × g, and the pellet was stored at −20 °C for the subsequent DNA extraction and sequencing.

### Flow column experiment design

**Fig. 1:**
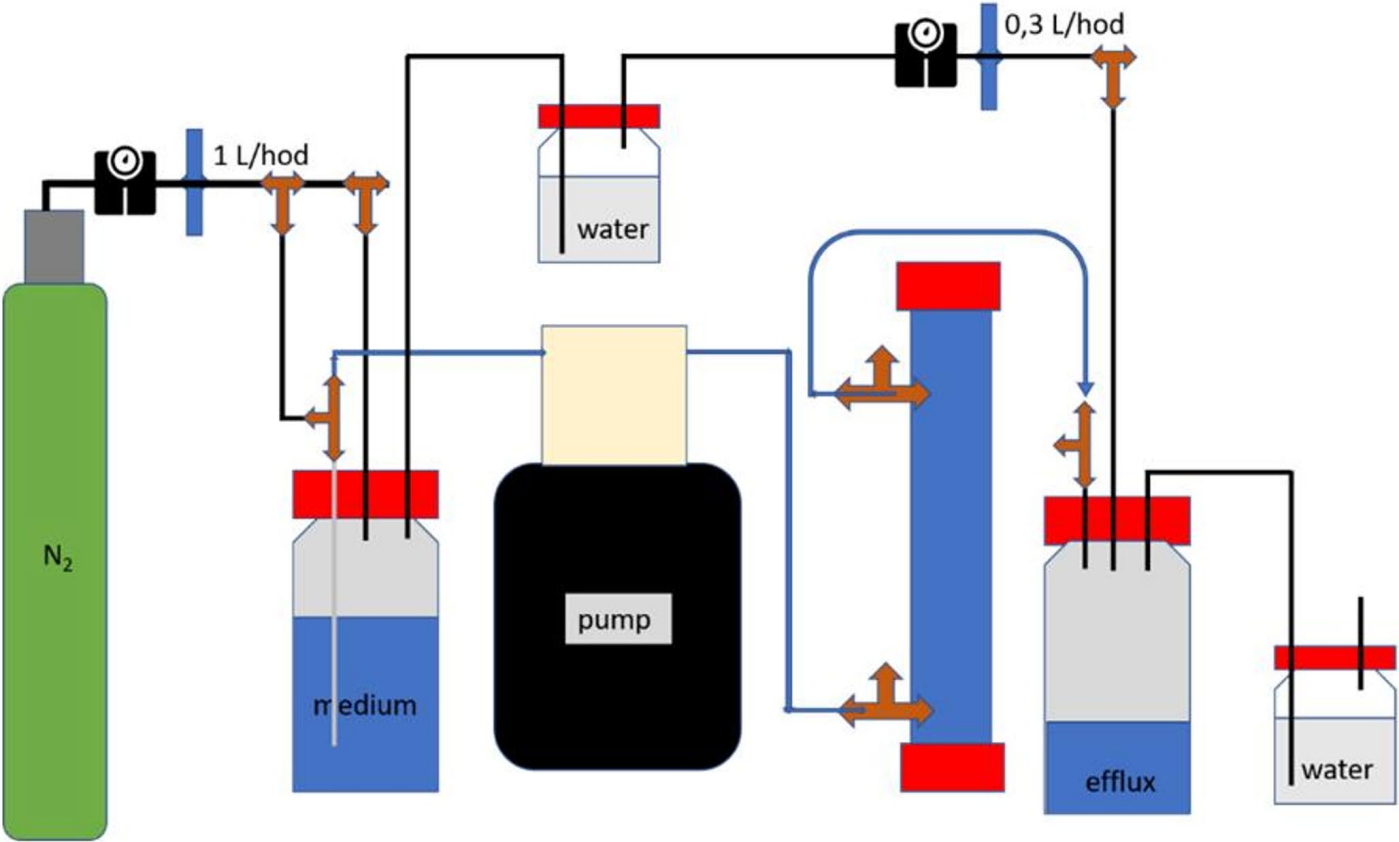
Schematic diagram of the flow-through column experiment

To simulate a worst-case scenario of corrosion processes in DGR with unrestricted nutrient access and simulating diffusion of metabolic products, continuous removal of corrosion products, and thus potentially higher corrosion rates (An et al. 2021), we conducted additional experiments in a dynamic flow system using two media types, NRM and R2A, both with 25% organic content. These two media were selected because significantly increased corrosion rates compared to sterile controls were observed for R2A25, while NRM25 promoted localized corrosion. Although the highest corrosion rates were often recorded in zero-organic (0%) media, these conditions yielded too low microbial abundance for reliable microbial community characterization. Reduced organic content (25%) was therefore selected as a compromise to limit the availability of alternative electron donors and promote electron uptake from metallic iron (EMIC), while still supporting sufficient microbial proliferation for reproducible molecular and surface analyses. A schematic of the dynamic flow system setup (shown for a single column for simplicity) is presented in Fig. 1. For each medium, eight sterile glass columns filled with eight corrosion coupons embedded in sterile glass beads (3 mm diameter) were prepared; four for flow and four for static conditions, including two sterile controls per condition. Each column had inlet and outlet ports for tubing connection and a rubber-sealed injection port for anoxic, sterile inoculation using a needle. A glass filter at the base of the column prevented backflow and clogging. Each column was connected to a stock media reservoir and an efflux bottle, with a peristaltic pump maintaining a bottom-up, unidirectional flow of approximately 40 mL per day for each column (equal to one column volume), ensuring a uniform stream of liquid from bottom to top. The columns were incubated at 30 °C. Media stock and efflux bottles were maintained under a continuous nitrogen flow with slight overpressure to prevent oxygen ingress. On day 0, the system was assembled, flushed with N₂, and filled with anoxic medium using the pump. The flow was then paused, and each column was inoculated with 2.5 mL of a 1-week-old bentonite preculture in the same medium (2 g bentonite in 50 mL medium). Following three days of static incubation, to promote microbial attachment to metal surfaces, allow adaptation to the new environment, and prevent cell washout, the flow was resumed and maintained continuously for 21 days. Parallel flow column experiments were run without flow (= static).

Post-experiment sample processing was as follows: 5 mL of medium was sampled from each column to analyze microbial composition. The sample was centrifuged at 11,000 × g, and the resulting pellet was stored at −20 °C for subsequent DNA extraction and 16 S rRNA amplicon sequencing. Corrosion coupons were then carefully removed from the column. In the inoculated columns, three coupons were swabbed to assess biofilm community analysis and then left to dry under anoxic conditions. The remaining coupons were dried for corrosion analysis. In sterile control columns, no swabbing was performed, and all coupons were left to dry for subsequent corrosion assessment.

### DNA Extraction

Cell pellets from liquid cultures were subjected to DNA extraction using the DNeasy PowerWater Kit (Qiagen, Germany) following the manufacturer’s protocol. Negative control samples (blank isolation controls) were included in every DNA extraction round to monitor potential contamination. Extracted DNA was quantified using the Qubit^®^ 2.0 Fluorometer (Invitrogen, Life Technologies, USA) and used for 16 S rRNA gene amplicon sequencing to reveal microbial composition of the samples.

### Relative quantification by qPCR

Changes in the relative abundance of total bacterial biomass were monitored using qPCR targeting the 16 S rRNA gene on a LightCycler^®^ 480 system (Roche, Switzerland). Universal primers U16SRT-F (5′-ACTCCTACGGGAGGCAGCAGT-3′) and U16SRT-R (5′-ATTACCGCGGCTGCTGGC-3′) targeting the V3 region were used (Clifford et al. 2012). Reaction setup and cycling conditions followed (Shrestha et al. 2022), with two technical replicates per sample; samples with replicate Cq differences > 0.5 were reanalyzed. Negative (no template) and positive controls were included in each run to verify detection limits and reproducibility. Because absolute quantification in complex environmental samples can be biased (Gaby And Buckley 2017), relative quantification (RQ) was applied using the ΔCq method (Lhotský et al. 2021; Shrestha et al. 2021), calculating fold changes in bacterial 16 S rRNA gene copy numbers between the studied samples and the corresponding medium sterile control.

### 16 S rRNA gene sequencing

Samples were categorized based on DNA yield and qPCR Cq values. For samples with low DNA concentration (< 0.5 ng·µL^− 1^) and high Cq (> 18), a two-step PCR was performed using universal and barcoded fusion primers 515 F (Dowd et al. 2008) and 802R (Claesson et al. 2010) targeting the hypervariable V4 region of the 16 S rRNA gene of both Bacteria and Archaea. Samples with sufficient DNA (> 0.5 ng·µL^− 1^ and Cq < 18) were amplified using a single PCR step with barcoded primers. PCR conditions included initial denaturation at 95 °C for 3 min, followed by 10 cycles (in the first PCR) or 35 cycles (in the second PCR) of denaturation at 98 °C for 20 s, annealing at 50 °C for 15 s, and elongation at 72 °C for 45 s, with a final extension at 72 °C for 1 min. A high-fidelity polymerase (EliZyme HS HIFI MIX, Elizabeth Pharmacon, Czech Republic) was used to minimize PCR-induced sequencing errors. Amplicons (< 400 bp) were purified using the Agencourt AMPure XP system with a 1:1 bead-to-sample ratio, and concentrations were quantified via Qubit^®^ 2.0. Equal concentrations of barcode-tagged amplicons (25 nM each) were pooled and sequenced using the Genexus Ion Torrent System (Thermo Fisher Scientific, USA) with the ION GX5 Chips.

### Bioinformatic processing

Raw sequencing reads were processed in QIIME 2 v2023.2 (Bolyen et al. 2019). Demultiplexing and quality filtering were conducted using the q2-demux plugin. Denoising, chimera removal, and generation of amplicon sequence variants (ASVs) were performed with DADA2 (Callahan et al. 2016). Taxonomic assignment was carried out using a naïve Bayes classifier (q2-feature-classifier) trained on the Silva 138 database (Quast et al. 2013), targeting the V4 region. Reads classified as chloroplast or mitochondrial were removed prior to analysis. To assess classification accuracy, ZymoBIOMICS Microbial Community DNA Standard (Zymo Research) was used as a reference. The final ASV table was imported into the R environment and analyzed with the phyloseq package (McMurdie and Holmes 2013). ASVs were aggregated to the genus level, and taxonomy bar chart plots were created to display genera with a mean relative abundance > 0.01. Lastly, statistical identification and removal of contaminant sequences was undertaken using the Decontam package v1.20.0 (Davis et al. 2018), based on DNA concentration and sequenced negative kit control samples (combined method option). The list of identified contaminants detected is presented in Supplementary Material 2, Supplementary Table S2.

### Statistical analyses

Differences in corrosion rates between sterile and inoculated samples, between Experiment 1 and Experiment 2 in the Batch experiment, and between dynamic and static conditions in the Flow column experiment were assessed using the Welch Two-Sample *t*-test for normally distributed data or the Wilcoxon rank-sum test for non-normally distributed data. These tests were conducted both globally and separately for each medium type. Similar tests were also applied to identify the differences in microbial relative quantity in the Batch experiment samples with different organic content. R was used for all statistical analyses.

Microbial community differences among treatments were visualized using principal coordinate analysis (PCoA) based on Bray–Curtis dissimilarity. To identify factors shaping microbial diversity and to assess the significance of differences between inferred groups, permutational ANOVA (PERMANOVA) was performed using the adonis function from the vegan R package (Martinez Abizu 2020). Observed alpha diversities were estimated with the phyloseq package (McMurdie and Holmes 2013) using R. We further performed correlation analysis linking microbial abundance with elevated corrosion rates across Batch experiments using the Spearman correlation coefficient in R.

To identify genera enriched explicitly on the coupon surface, which may represent microorganisms involved in microbiologically influenced corrosion (MIC), we identified the samples with the highest corrosion rates across different media and experiments. We compared the microbial composition of the corresponding bulk culture for these samples with the swab taken directly from the corrosion coupons. We focused on genera with a mean relative abundance greater than 5% across compared swab and bulk medium samples, calculated their average frequency, and estimated the deviation from the mean value to highlight those preferentially associated with the corrosion coupon surface.

### Corrosion analyses

Corrosion processes on the coupon´s surface during exposures were evaluated by weight loss (1601 MP8, Sartorius, Germany), phase composition of corrosion products (XRD, X’Pert Pro, PANalytical, EA Almelo, Holland) and observing the morphology of the surface by optical stereomicroscope (SZX 10, Olympus, Japan), scanning electron microscope (VEGA3 LMU, TESCAN, Czech Republic), and digital microscope with surface profile measurement (VH-Z250R, Keyence, Japan). The acquisition of SEM images was achieved using tungsten cathode with a secondary electron and backscattered electron (BSE) detector under an accelerating voltage 20 kV and an approximate zoom of 500x. The digital microscope measures surface profiles by scanning the sample at multiple focus levels (Z-axis). The system detects the height at which each pixel is in focus to create a 3D height map. From this, the software generates a height profile along any selected line for precise dimensional or roughness analysis. The measurement was performed at magnifications of 100x – 250x.

The treatment of the carbon steel coupons after exposure to microbes was carried out in the following steps: documentation of the surface with an optical stereomicroscope, analysis phase composition of the corrosion products, and pickling (chemical removal) of the corrosion products accoridng to standard ISO 8407 in a solution of distilled water (500 mL), concentrated hydrochloric acid (500 mL) and hexamethylenetetramine (HMTA) (3.5 g). After pickling, the sample was rinsed with distilled water and ethanol. Clean and dry sample was weighted on analytical balance (1601 MP8, Sartorius, Germany).

Scanning electron microscope and digital confocal microscope with surface profile measurement were employed individually according to weight loss results or when some important characteristic features were observed on the surface.

## Results

### Batch experiments

#### Corrosion results

The observed corrosion losses varied depending on the type of experimental setup and between individual experiments. Because the corrosion attack was not strictly uniform, corrosion rates based on weight loss are expressed in g·m^2^·a^− 1^, but these values are for better comparison with the literature also recalculated to µm·a^− 1^.

**Fig. 2:**
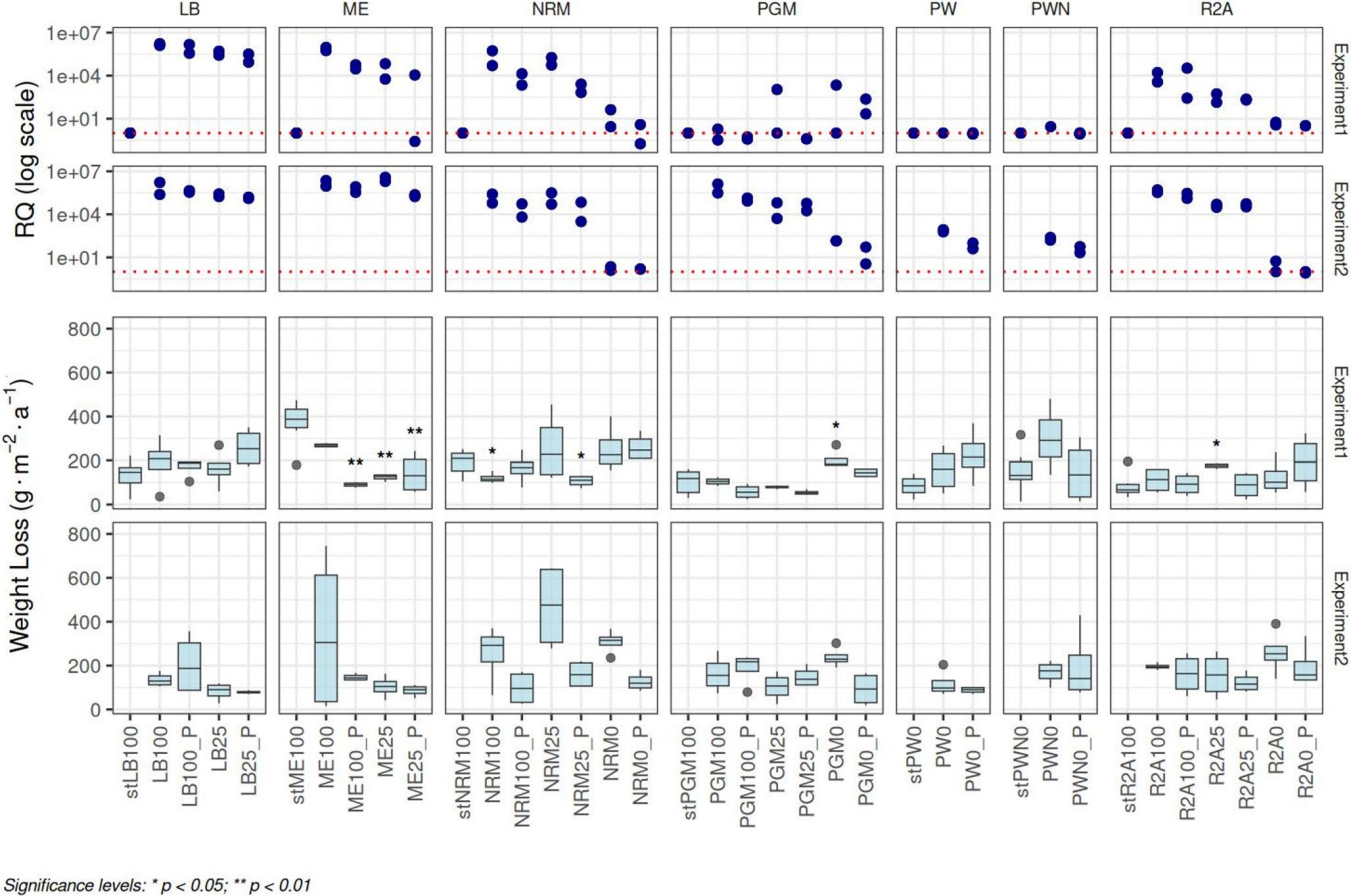
Relative quantities of 16 S rRNA gene copies compared to sterile controls (RQ) and corrosion mass losses (g·m^−2^·a^−1^) in different sample types studied in Experiments 1 and 2. The dashed line in the RQ graphs indicates the level corresponding to sterile samples. Asterisks indicate treatments with corrosion losses that are significantly different from sterile controls in Experiment 1

In LB, PW, and PNW media, the peak corrosion rates occurred during Batch experiment 1, reaching 257 g·m⁻²·a⁻¹ (33 μm·a⁻¹), 220 g·m⁻²·a⁻¹ (28 μm·a⁻¹), and 307 g·m⁻²·a⁻¹ (39 μm·a⁻¹), respectively, Fig. 2. Conversely, the highest rates in NRM, PGM, and R2A media were measured during Experiment 2, with 468 g·m⁻²·a⁻¹ (60 μm·a⁻¹), 238 g·m⁻²·a⁻¹ (31 μm·a⁻¹), and 259 g·m⁻²·a⁻¹ (33 μm·a⁻¹) values, respectively (Fig. 2). Notably, the ME medium exhibited its highest corrosion rate and the second-highest overall rate across all experiments in the sterile control samples (369 g·m⁻²·a⁻¹ corresponding to 47 μm·a⁻¹) and was the only medium where sterile controls showed higher corrosion rates than inoculated samples (Fig. 2). When evaluating relative corrosion losses compared to abiotic controls, the most pronounced increases were observed in R2A (3-fold), PW (2.6-fold), NRM (2.4-fold), and PGM (2.3-fold) media. Except for PW, all these were detected in Experiment 2.

Although certain trends were observed in corrosion rates across both abiotic controls and inoculated samples, the differences were not statistically significant for individual media types in most cases (Fig. 2; Supplementary Material 2, Supplementary Table S3). Similarly, differences in corrosion rates between Experiments 1 and 2 were observed in some cases but not statistically significant, either globally (Wilcoxon signed-rank test, *p* = 0.505) or within specific media types (Supplementary Material 2, Supplementary Table S4) except for R2A medium, which showed weakly significant differences between both Experiments (paired *t*-test, *p* = 0.048). This lack of significance is likely attributable to the limited number of coupons per sample and relatively high variability in the measured data.

To further explore the potential microbial contribution to corrosion, we examined the relationship between relative microbial quantity (estimated from relative increase in 16 S rRNA gene copy numbers (RQ) compared to corresponding medium sterile controls) and corrosion rate. Spearman correlation analysis revealed no significant association between microbial quantity and corrosion rate in either the full dataset (*p* = 0.516, *r* = 0.0552) or in the biotic samples only (*p* = 0.962, *r* = 0.0044). In accordance to that, microbial quantity (RQ) increased with organic content and all pairwise differences between organic groups in relative abundance were significant (0 vs. 25%: *p* = 1.42 × 10⁻⁹, 0 vs. 100%: *p* = 1.95 × 10⁻¹⁰, 25 vs. 100%: *p* = 0.0112) but an opposite trend was observed for corrosion rates (Supplementary Material 1, Supplementary Figure S1), with significantly higher corrosion rates in samples with 0% than in 25% organic content (*p* = 0.0045), while samples with 0% vs. 100% and 25% vs. 100% were not significantly different. These findings suggest that while organic content strongly influences microbial abundance, its impact on corrosion rate is more limited and does not follow the same trend.

**Fig. 3:**
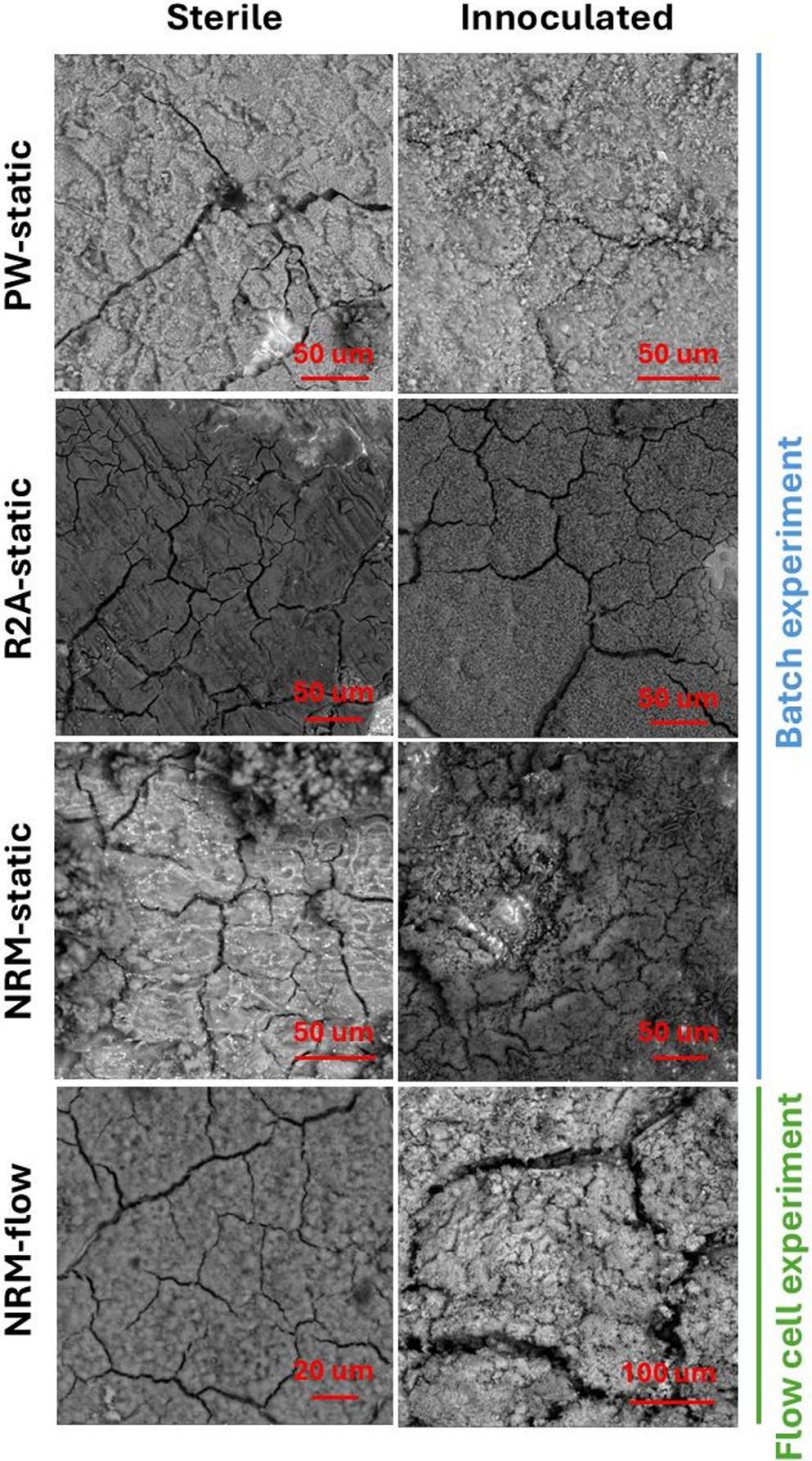
SEM images of the surface after exposure to different culture media in Batch and Flow column experiments: bentonite pore water (PW, static), R2A (static), and NRM under static and dynamic flow conditions

**Fig. 4:**
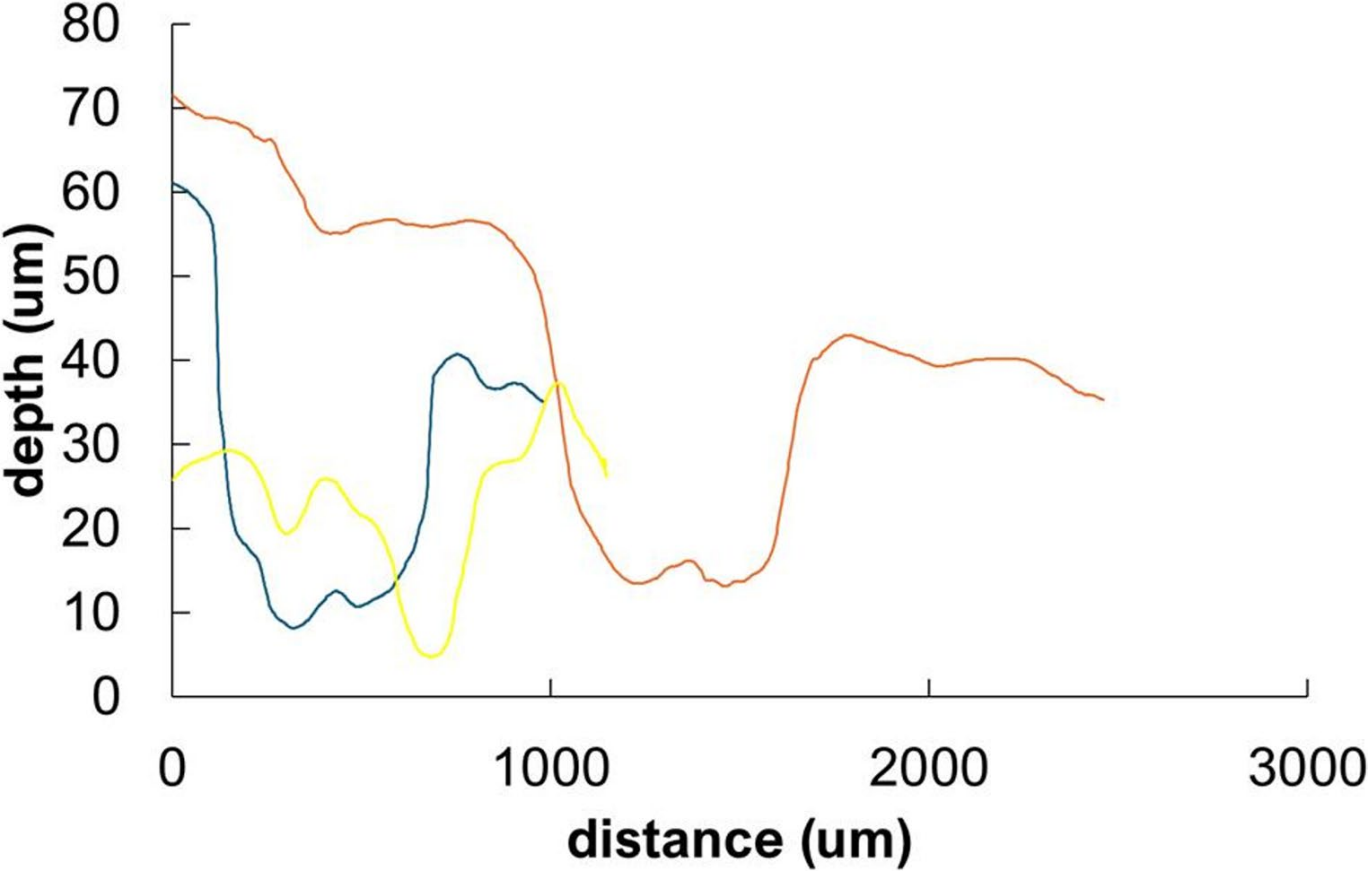
Examples of optical profilometry scans of the pits formed after exposure in NRM medium. The largest pits exhibit diameters of up to 600 μm and a depth of approximately 40 μm

The morphology of the corrosion products corresponds to the observed corrosion patterns described above. In sterile pore water (PW) and R2A, the corrosion product layer is relatively homogeneous and thus likely provides effective protection against further corrosion (Fig. 3), resulting in approximately half the corrosion rate under sterile conditions compared to sterile NRM, which forms coarse crystalline corrosion products. Nevertheless, this homogeneous corrosion product layer does not represent a limiting barrier under biotic conditions, where the corrosion rates increase (Fig. 2). Finally, the biotic NRM medium produces an even more heterogeneous layer, characterized by a mixture of crystal types distributed across the surface, and the spots covered with coarse crystals (Fig. 3). After pickling, deep corrosion pits were observed in these regions, with a density of 5 pits/cm^2,^ and optical profilometry confirmed a maximum penetration depth of nearly 40 μm (Fig. 4). This is notably higher than the average penetration depth of ~ 15 μm estimated from overall weight loss after three months of exposure in inoculated NRM samples. This corresponds to a localisation factor (ratio of maximal local penetration and average mass loss penetration) of 2.7. The presence of a heterogeneous layer composed of corrosion products and biofilm within the pits (Supplementary Material 1, Supplementary Figure S2) suggests a microbial contribution to pitting corrosion. These regions show pronounced enrichment in oxygen and carbon based on elemental mapping (Supplementary Material 1, Supplementary Figure S3). XRD analysis indicates that the corrosion products are dominated by oxo-hydroxides (goethite) with traces of croenstedtite (low concentration of silicates from bentonite used as innoculum) (Supplementary Material 1, Supplementary Figure S4). In the absence of carbonates in the solution (Table 1), the detected carbon is attributed to organic matter.

Among the tested media, NRM exhibited the highest corrosion rates together with pronounced localized attack. In R2A25, corrosion rates in inoculated samples were significantly higher than in sterile controls. On this basis, NRM25 and R2A25 were selected for subsequent flow experiments.

#### Microbial composition in medium and swab samples

The DNA extraction of the abiotic control samples resulted in DNA concentration below the Qubit detection limit and thus confirmed the sterility of the control samples. The degree of corrosion found in the control samples can, therefore, be attributed purely to abiotic corrosion.

Only very low microbial diversity and very low read counts were observed in the kit controls co-extracted with samples during each DNA extraction after Decontam treatment, and are visualized in Supplementary Material 1, Supplementary Figure S5. Kit controls were thus not involved in further analyses, and the potential effect of contaminants was considered negligible for the following data analyses.

To interpret the microbial composition data, we first performed a PERMANOVA incorporating various experimental factors that could influence community diversity. Results are summarized in Table 3. The analysis revealed that all tested factors significantly contributed to shaping microbial diversity, although their relative impacts varied. The most influential factor was the type of growth medium, which accounted for 22.5% of the observed variability. This was followed by the level of organic content (0%, 25%, or 100%), which explained 6.5% of the variation. In contrast, sample type (swab vs. medium) and experimental batch (Experiment 1 vs. 2) had less pronounced effects, explaining 2.6% and 1.8% of the variability, respectively. Interestingly, the inclusion of iron powder had only a minor influence, accounting for just 0.6% of the variation in microbial composition.

**Table 3:** Results of PERMANOVA analysis testing the effect of various experimental factors on differences in microbial composition between the tested groups. *** *p* ≤ 0.001, ** *p* ≤ 0.01, * *p* ≤ 0.05

	Df	SumOfSqs	R2	F	Pr(> F)	significance
Medium type	5	15.116	0.22539	12.3621	0.001	***
Organics content	2	4.353	0.06490	8.8991	0.001	***
Sample type (swab x medium)	1	1.728	0.02577	7.0668	0.001	***
Experiment (1 or 2)	1	1.184	0.01766	4.8424	0.001	***
Powder Fe content	1	0.422	0.00629	1.7237	0.026	*

Correspondingly, principal component analysis (PCoA) revealed that microbial composition varied mainly with medium type and organic content. While some overlap occurred, most media supported distinct communities forming well-separated clusters (Supplementary Material 1, Supplementary Figure S6). Samples with 0% organic content consistently showed reduced biomass and clustered separately, underscoring the limiting role of organic matter in microbial growth.

**Fig. 5:**
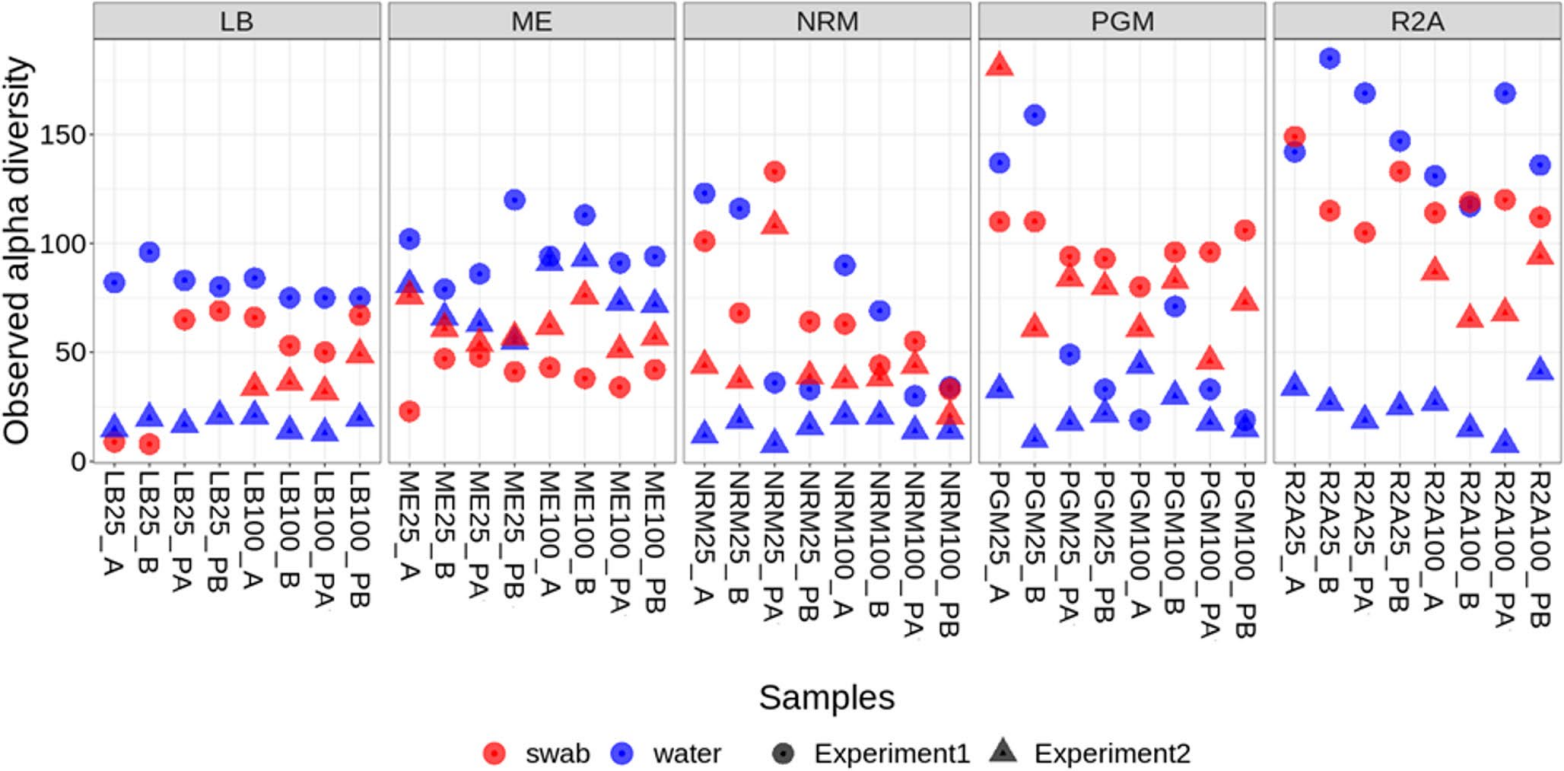
Observed alpha diversity in bulk media and swab samples from respective media with 25% and 100% of organic content compared to the original prescription. LB - Lysogeny Broth, ME - medium 120 (Koblitz et al. 2023), NRM - Nitrate Broth, PGM - Postgate medium, R2A - R2A medium; 100/25–100%/25% of organic content compared to the original prescription; A/B - replicate samples; P - samples containing powdered iron

To characterize overall diversity patterns across cultivation conditions, alpha diversity was further evaluated for both bulk media and swab samples and compared between Experiments 1 and 2. Non-organic treatments were excluded from this analysis due to the absence of swab samples and inconsistent microbial community composition (Fig. 5). In bulk media samples, Experiment 1 generally showed the highest alpha diversity across most media and sample types compared to Experiment 2, with the exception of most PGM samples and some NRM samples. In contrast, bulk media samples from Experiment 2 exhibited the lowest diversity across all media, except for ME, where alpha diversity was slightly higher than in Experiment 1. A similar pattern was observed in swab samples: diversity was generally higher in Experiment 1 than in Experiment 2 across all media, again with the exception of ME. However, the difference in diversity between experiments was less pronounced in swab samples than in bulk media samples. When comparing swab versus bulk media samples within each experiment, bulk media samples in Experiment 1 exhibited higher diversity than swabs across all media except PGM, where the pattern is likely biased by sequencing issues affecting bulk medium samples in Experiment 1. In Experiment 2, this relationship was reversed: swab samples generally displayed higher diversity than bulk media samples, except in ME. Notably, ME was the only medium in which bulk media diversity remained consistently high across both experiments and in which swab diversity remained relatively stable between experiments. The observed alpha diversity visualization further showed that for most media types, higher diversity was observed in media with reduced organic content (25%) compared to full media (Fig. 5), which supported the choice of media with 25% organic content for the following flow cell experiment.

**Fig. 6:**
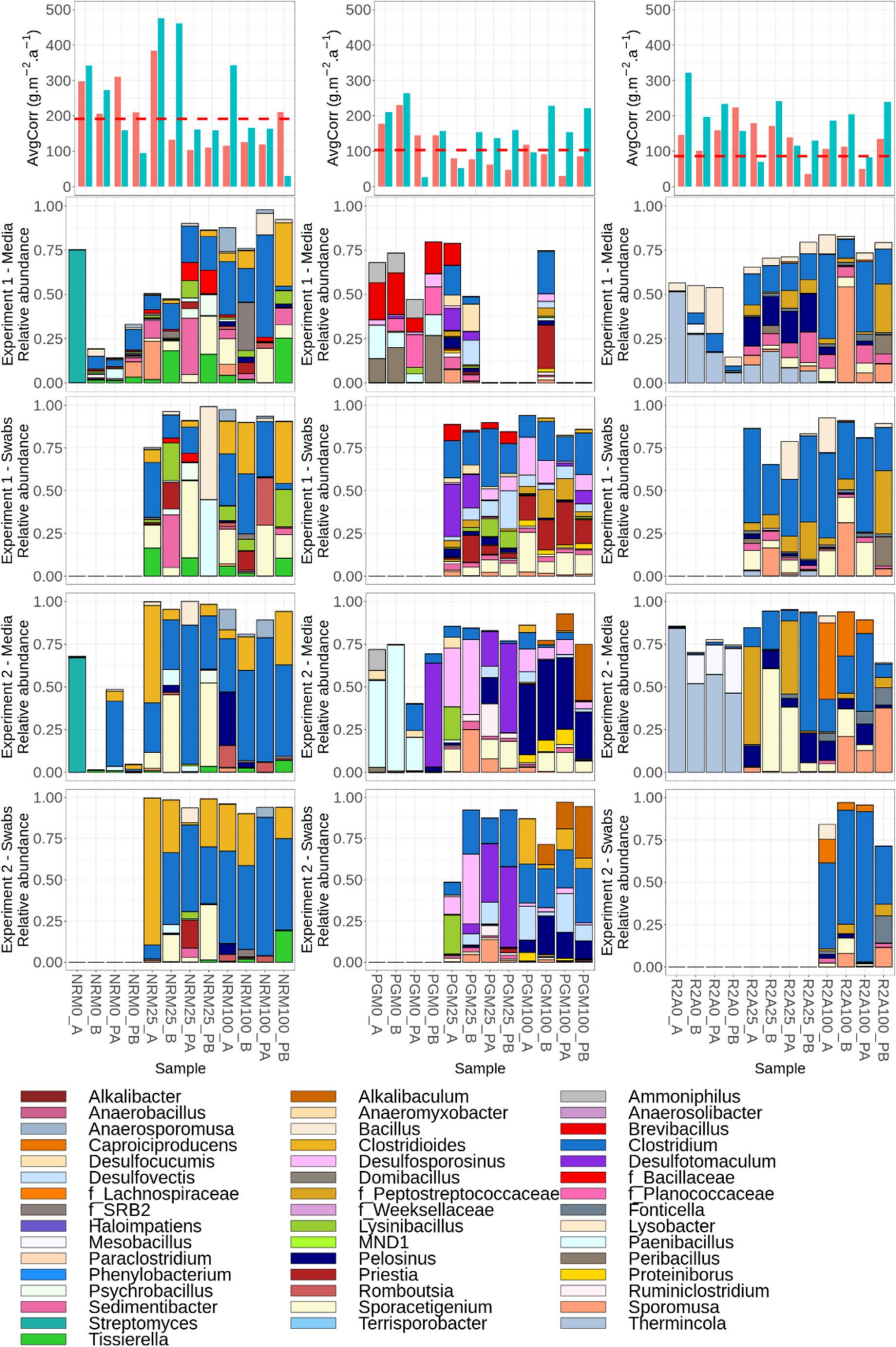
Mean corrosion rates (AvgCorr) in g·m^−2^·a^−1^ and microbial composition detected in individual samples studied. AvgCorr graphs - red and blue bars represent mean corrosion rate of corresponding samples in Experiment 1 and 2 respectively (error bars are not shown due to only two coupons per condition); the dashed red line indicates the mean corrosion rate in the abiotic controls; PW - synthetic bentonite pore water, PWN - PW amended with nitrate, PGM - Postgate medium, LB - Lysogeny Broth, NRM - Nitrate Broth, R2A - R2A medium, ME - medium 120 (Koblitz et al. 2023); 100/25/0–100%/25%/0% of organic content compared to the original prescription; Numbers 1 and 2 indicate replicates; P - powdered iron included in the sample

**Fig. 7:**
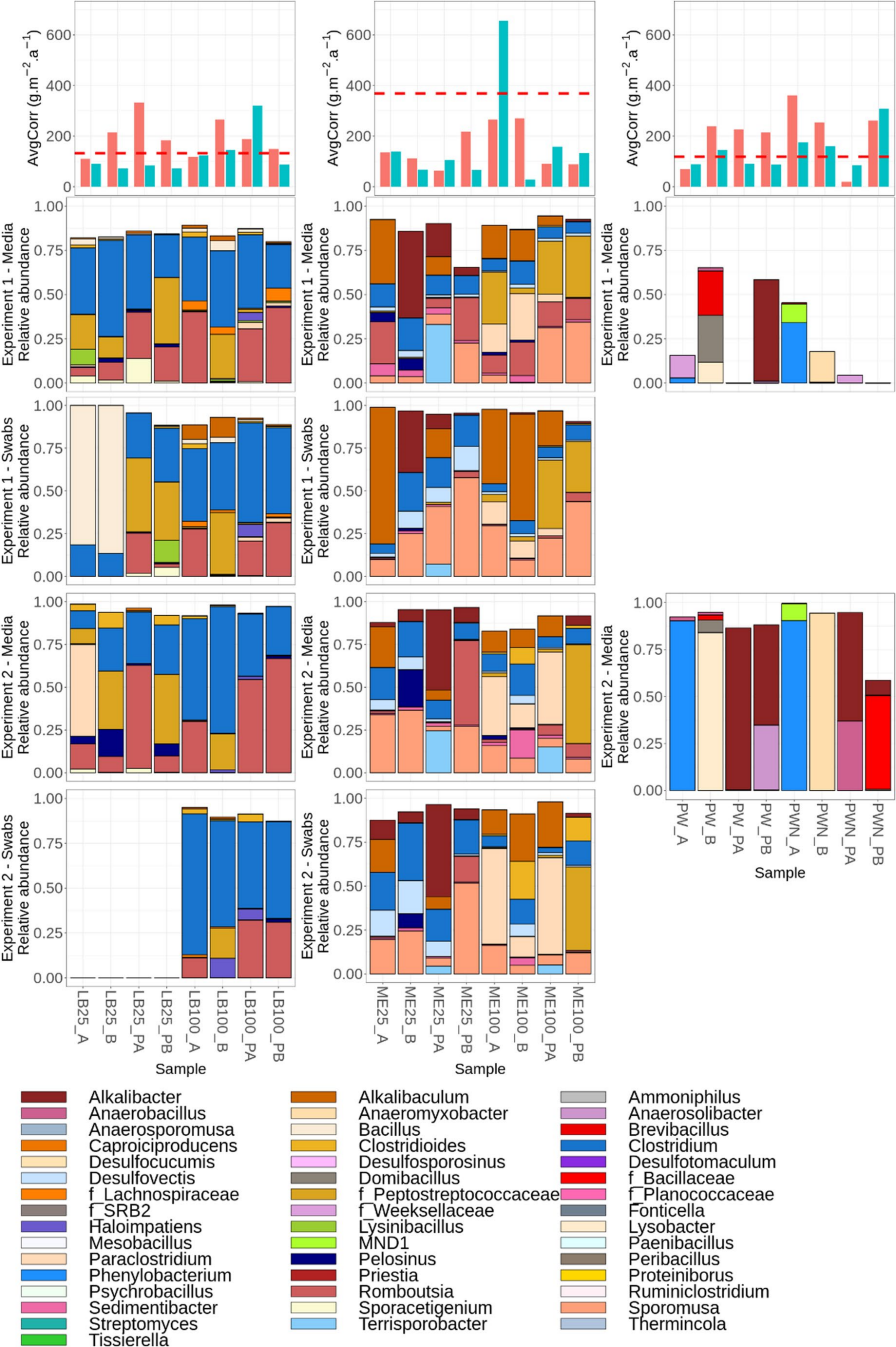
Mean corrosion rates (AvgCorr) in g·m-2·a-1 and microbial composition detected in individual samples studied. AvgCorr graphs - red and blue bars represent mean corrosion rate of corresponding samples in Experiment 1 and 2 respectively (error bars are not shown due to only two coupons per condition); the dashed red line indicates the mean corrosion rate in the abiotic controls; PW - synthetic bentonite pore water, PWN - PW amended with nitrate, PGM - Postgate medium, LB - Lysogeny Broth, NRM - Nitrate Broth, R2A - R2A medium, ME - medium 120 (Koblitz et al. 2023); 100/25/0–100%/25%/0% of organic content compared to the original prescription; A/B - replicate samples; P - samples containing powdered iron. LB25 swab samples were not analysed in Experiment 2 due to sampling error

The microbial composition of the inoculated samples, as well as the corresponding corrosion rates, are summarized in Figs. 6 and 7. Swab samples were analyzed only for media with 25% and 100% organic content, as 0% media generally did not yield sufficient biomass for reliable genetic analysis.

NRM media samples displayed a diverse microbial composition, especially in Experiment 1. In NRM25 (25% organic content) bulk medium samples, *Sporomusa*, *Tissierella*, *Sedimentibacter*, *Sporoacetigenium*, and *Clostridium* were detected, with *Sporoacetigenium* and *Clostridium* abundant in all samples, while the other genera occurred sporadically. In NRM100 bulk medium samples, *Clostridium*, *Sporoacetigenium*, *Clostridioides*, *Tissierella*, and a taxon f_SRB2 were observed, with *Clostridium* and *Clostridioides* present in most samples. In Experiment 2, *Clostridium* and *Clostridioides* or *Sporoacetigenium* dominated most samples across all organic content levels, suggesting active fermentative metabolism. Swab samples in Experiment 1 again showed a high number of taxa, only partially reflecting the trends in the bulk cultures. Notably, the number of dominant taxa increased with decreasing organic content, and NRM genus *Lysinibacillus* and fermentative *Clostridioides* and acetogenic *Sporoacetogenium* tend to be enriched explicitly on the corrosion surface. In NRM0 samples (0% organic content), *Streptomyces* dominated the NRM0_A bulk medium sample in both experiments. The NRM0_B bulk medium samples showed weak signals, with *Clostridium* and *Clostridioides* identified in one sample from Experiment 2.

In the PGM culture medium, Experiment 1, despite high microbial abundance inferred from swab DNA yields, amplification from liquid cultures was inefficient, likely due to the presence of inhibitors in the media (presumably ferrous ions (Bonsu et al. 2024). PGM0 bulk medium samples (no organic content) were dominated by Bacilli. In Experiment 1, these included *Peribacillus*, *Brevibacillu*s, and *Paenibacillus*, while in Experiment 2, *Paenibacillus* predominated, although *Desulfotomaculum* also dominated one sample. PGM25 bulk media samples (25% organic content) that could be sequenced contained diverse sulfate-reducing bacteria (SRB), including *Desulfocucumis*, *Desulfotomaculum*, and *Desulfovectis* in Experiment 1, and *Desulfotomaculum* or *Desulfosporosinus* in Experiment 2, depending on powder iron presence. For the fully organic PGM100 bulk media samples, only one sample (PGM100_2) was successfully sequenced in Experiment 1. In Experiment 2, sequencing was more successful, and all samples were dominated by *Pelosinus* and *Desulfosporosinus*. Swab samples showed more consistent patterns. In Experiment 1, PGM25 swabs were dominated by *Desulfovectis*, *Desulfotomaculum*, and *Clostridium*, while PGM100 swabs contained *Priestia*, *Clostridium*, and *Desulfosporosinus*. In Experiment 2, PGM25 swabs showed dominance of either *Desulfotomaculum* or *Desulfosporosinus* depending on iron content, whereas PGM100 swabs contained *Clostridium*, *Pelosinus*, and *Alcalibaculum*, with additional less abundant SRB taxa present.

In the case of R2A medium, R2A0 (0% organic content) bulk media samples in Experiment 1 were dominated by *Thermincola*,* Bacillus*, and *Clostridium*. In Experiment 2, *Thermincola* and *Mesobacillus* prevailed. In R2A25 (25% organic content), microbial communities of bulk media included *Clostridium*, *Pelosinus*, and *Thermincola* in Experiment 1, and shifted toward *Sporacetigenium*, *Clostridium*, and *Peptostreptococcaceae* in Experiment 2, indicating anaerobic fermentation under moderate nutrient levels. In R2A100 (fully organic), *Clostridium*, *Sporomusa*, and *Caproiciproducens* were predominant in bulk media, suggesting fermentative anaerobic metabolism. Swab samples showed an elevated abundance of *Clostridium* but lacked *Caproiciproducens*, which dominated corresponding cultures.

In LB25 and LB100 (25% and 100% organic content), bulk media samples in Experiment 1 were dominated by *Clostridium*, together with either *Rhomboutsia* or an unspecified member of the family Peptostreptococcaceae. In Experiment 2, the pattern changed only for LB25 media samples, where *Rhomboutsia*, members of Peptostreptococcaceae, and in one sample *Paraclostridium* tended to increase in relative abundance. Swab samples generally reflected the composition of the bulk culture samples. An exception was observed in LB25 swabs from the samples without powder iron in Experiment 1, where *Bacillus* dominated despite being a minor component in the bulk media samples. However, this divergence did not correspond to increased corrosion. Swab samples from LB25 in Experiment 2 were not collected due to sampling error.

The ME medium, which was intended to target methanogens, supported variable and relatively diverse, predominantly fermentative microbial communities in both experiments. However, no methanogens were detected, indicating that the applied cultivation conditions were not suitable for methanogenic enrichment. Instead, the ME media samples were dominated by diverse acetogenic and fermentative taxa. In Experiment 1, the dominant genera in ME25 medium (25% organic content) in bulk media were *Romboutsia*, *Alkalibacter*, and *Alcalibaculum*, whereas in Experiment 2, *Sporomusa* became more abundant. In ME100 (fully organic) bulk media samples, Peptostreptococcaceae, *Sporomusa*, *Alcalibaculum*, and *Paraclostridium* dominated in Experiment 1. In Experiment 2, *Paraclostridium* increased while Peptostreptococcaceae declined. Swabs showed similar trends: in Experiment 1, ME25 swabs were dominated by *Clostridium* and *Sporomusa*, while ME100 swabs were enriched in *Alcalibaculum*, *Paraclostridium*, and *Clostridioides*. In Experiment 2, similar community members were observed, with a higher number of dominant taxa in swabbed biofilms.

Finally, samples cultivated in PW and PWN media, which contain no added organic carbon and thus very low organic content, displayed low and stochastic microbial composition in bulk media in Experiment 1. In Experiment 2, each sample was typically dominated by one or two genera, with the community composition varying according to sample composition. PW bulk media samples containing powdered iron were dominated by *Alkalibacter*, whereas other samples were dominated by different genera such as *Lysobacter*, *Phenylobacterium*, *Anaerobacillus*, members of the family *Bacillaceae*, or *Anaeromyxobacter*. Swabs were not performed due to low overall biomass.

#### Identification of microbial taxa potentially involved in MIC

Initially, we conducted a correlation analysis to investigate potential links between overall microbial diversity and elevated corrosion rates across our Batch experiments. The goal was to identify taxa whose presence correlated with increased corrosion. However, due to the high variability in microbial community composition across individual samples resulting from many different media types and also a limited number of coupons and sample replicates, the analysis did not yield any statistically significant associations.

Considering these limitations, we decided to rely on an alternative approach, and we compared the microbial composition of the bulk medium samples with that of the corresponding swab samples taken directly from the coupon surfaces. PCoA analysis (Supplementary Material 1, Figure S3) showed poorly resolved clustering and no clear separation between bulk media and swab samples across most media types, indicating that swab-derived communities were generally compositionally similar to those obtained from bulk media. Although PERMANOVA detected a statistically significant difference between swab and bulk samples across all different media types, this factor explained only 2.6% of the total variability (Table 3). Correspondingly, pairwise PERMANOVA identified significant differences only for PGM (*p* = 0.001, R² = 10.7%) and R2A (*p* = 0.003, R² = 13.3%) media (Supplementary Material 2, Supplementary Table S5).

**Fig. 8:**
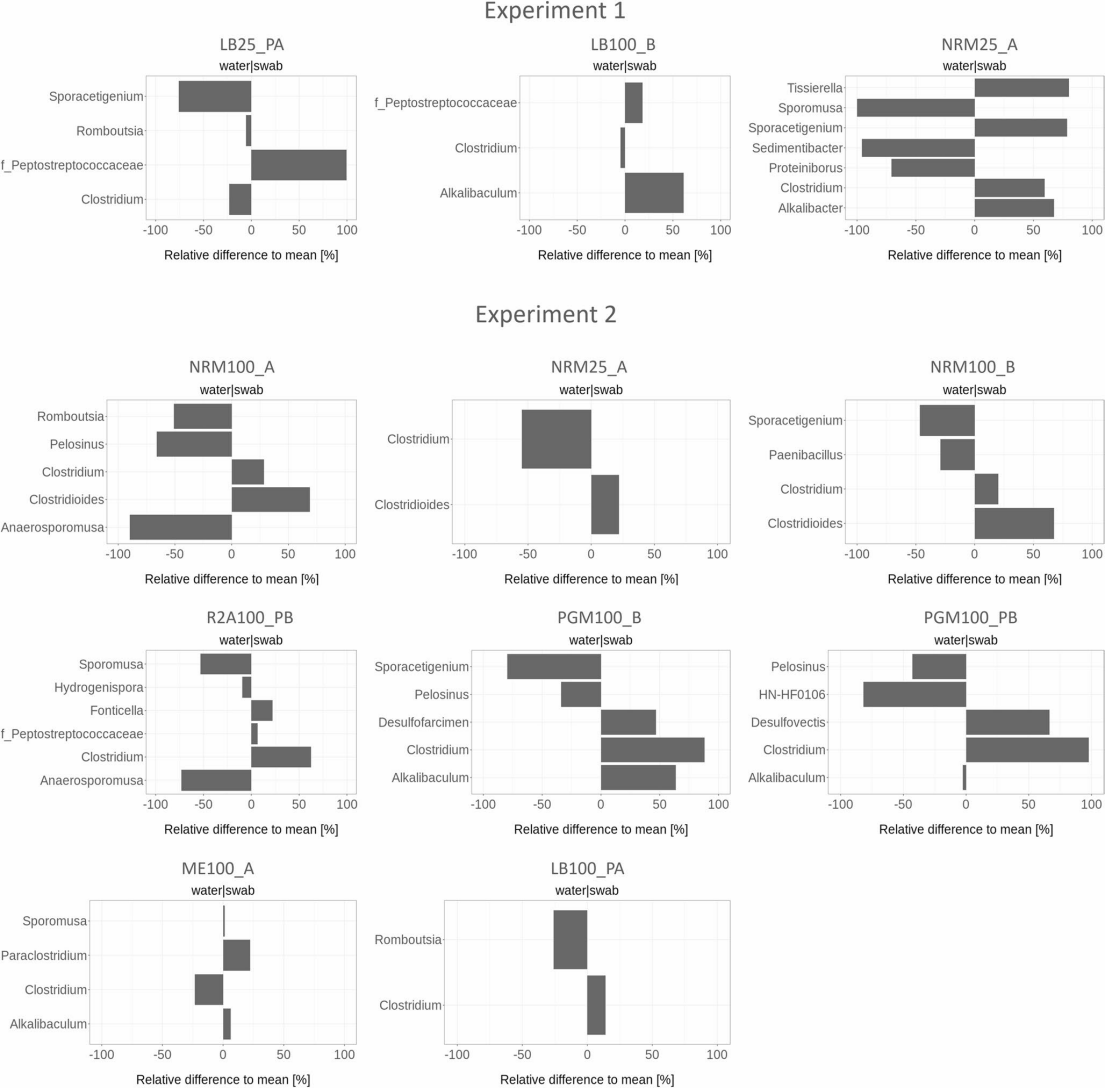
Relative differences (%) from the mean abundances of dominant bacterial genera (≥ 5% mean relative frequency) are shown for each pair of bulk medium and the corresponding swab sample in Experiments 1 and 2, under the 11 most corrosive conditions as determined by weight loss analysis. PGM - Postgate medium, LB - Lysogeny Broth, NRM - Nitrate Broth, R2A - R2A medium, ME - medium 120 (Koblitz et al. 2023). 25/100 - Media with 25% and 100% organic content compared to the original prescription. A/B - replicate samples, P - samples containing powdered iron

Because the global comparison of bulk media and swab samples across all conditions did not reveal any consistent patterns, we subsequently focused on the 11 samples with the highest corrosion rates across both experiments (Figs. 6 and 7), for which both bulk media and swab composition data were available. In these samples, we estimated the relative differences (in %) from the mean abundances of dominant bacterial genera (≥ 5% mean relative frequency) to identify genera enriched explicitly in the swab communities. The results are summarized in Fig. 8.

In Experiment I, we observed an enrichment of the family *Peptostreptococcaceae* in the LB25 and LB100 samples and *Alkalibaculum* in the LB100 sample, while *Tissierella*, *Sporoacetogenium*, *Clostridium*, and *Alkalibacter* were enriched in the NRM25 swab sample (Fig. 8).

In Experiment 2, different media types resulted in distinct microbial compositions in swab samples, with several genera showing sample-specific enrichment patterns (Fig. 8). Nevertheless, the genus *Clostridium* and the taxon *Clostridioides* were frequently enriched in all NRM swab samples, which were associated with elevated corrosion rates. These taxa also dominated most other swab samples across multiple conditions. The only exception was the ME100 sample, where *Clostridium* and *Clostridioides* were not specifically enriched; instead, *Paraclostridium* and *Alkalibaculum* were predominant. Other genera displayed more sample-specific enrichment patterns. For instance, the R2A100 swab sample was enriched in *Clostridium*, *Fonticella*, and members of the family *Peptostreptococcaceae*, while the PGM100 swab samples showed enrichment of *Clostridium*, *Desulfofarcimen*, *Desulfovectis*, and *Alkalibaculum*.

Many samples with 0% organic content also exhibited high corrosion rates compared to the sterile samples. Although swabbing was not feasible in these samples due to generally low biomass, the genera detected in the culture-based samples (see above) can also be considered potentially corrosive and warrant further investigation. Here, we provide a brief taxonomic overview. Various Bacilli, such as *Peribacillus*,* Brevibacillus*, or *Paenibacillus*, consistently dominated PGM0 sample*s*. R2A0 cultures were dominated by *Thermincola*, *Bacillus*, *Clostridium*, and *Mesobacillus*. In NRM0 samples, we identified *Streptomyces*, *Clostridium*,* and Clostridioides*. PW and PWN media showed low and stochastic microbial composition, and we identified *Alkalibacter*, *Lysobacter*, *Phenylobacterium*, *Anaerobacillus*, or *Anaeromyxobacter* among the dominants. Several of the detected taxa have previously been associated with MIC in other systems.

## Flow column experiments

### Corrosion results

**Fig. 9:**
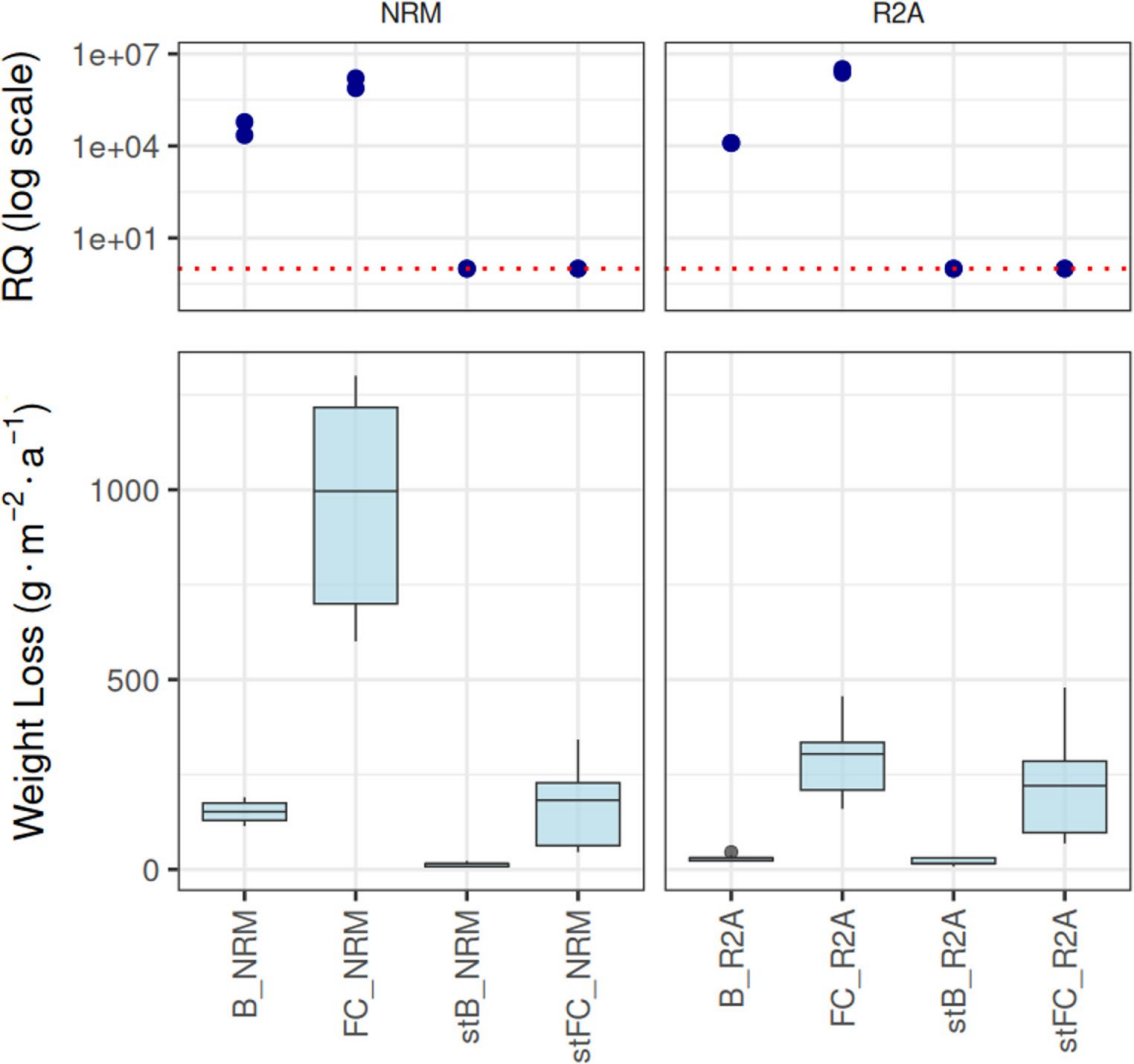
Relative quantities of 16S rRNA gene copies corresponding to sterile media samples (RQ) and corrosion mass losses (g·m^− 2^·a^− 1^) in individual flow column samples (each one with 8 corrosion coupons) studied. FC - dynamic flow columns, B - static flow columns, NRM - Nitrate Broth, R2A - R2A medium

The microbial relative abundance (based on 16 S rRNA gene copy numbers) differed between the treatments in the flow column experiments. We have observed aproximatelly 13.5 times higher relative abundance in dynamic (FC) conditions compared to stationary (B) in NRM medium and even 44 times higher abundance in R2A medium (Fig. 9), the differences for both media between biotic FC and B samples relative quantity were statistically significant (Welch Two Sample t-test, *p* = 0.021 for NRM and *p* = 2.93 × 10⁻³ for R2A).

Similarly, we observed differences in corrosion rates between both sterile and inoculated samples, as well as between dynamic flow and static systems. Under dynamic conditions, corrosion rates increased dramatically compared to static conditions in both sterile and inoculated samples for both media types (Fig. 9). In the NRM medium, dynamic sterile samples exhibited a mean corrosion loss of 159.7 g·m⁻²·a⁻¹ (21 μm·a^− 1^), compared to 14 g·m⁻²·a⁻¹ (2 μm·a^− 1^) in static sterile samples, which is a significant difference (Wilcoxon rank sum test, *p* = 5.923 × 10⁻⁶) and approximately 11.8-fold increase due to flow. Similarly, inoculated dynamic samples showed a mean corrosion loss of 971 g·m⁻²·a⁻¹ (125 μm·a^− 1^), compared to 150.5 g·m⁻²·a⁻¹ (19 μm·a^− 1^) in static inoculated samples, corresponding to a 6.5-fold increase and significant difference (Welch Two Sample t-test, *p* = 9.368 × 10⁻^8^). Furthermore, under dynamic flow conditions, corrosion rates in inoculated samples were approximately 6.1 times higher than in sterile samples, and the difference was again significant (Welch Two Sample t-test, *p* = 2.81 × 10⁻^8^). Under static conditions, this difference was even more pronounced, with inoculated samples exhibiting corrosion rates approximately 11.1 times higher than their sterile counterparts. Again, there was a significant difference (Wilcoxon rank sum test, *p* = 5.817 × 10⁻⁶). The results are summarized in Table 4. No corrosion localization was observed in this experiment’s inoculated NRM flow or static samples.

**Table 4:** Comparison of corrosion rates (g·m^− 2^·a^− 1^) between inoculated and sterile and static and flow samples in NRM and R2A media experiments. BIO - biotic factor, FLOW - dynamic flow factor. RST - Wilcoxon rank-sum test with continuity correction, TSTT - Welch’s two-sample t-test. NS - not significant, * = *p* < 0.05, ** = *p* < 0.005, *** = *p* < 0.0005

	R2A (g·m^− 2^·a^− 1^)	Fold increase due BIO		Fold increase due FLOW		NRM (g·m^− 2^·a^− 1^)	Fold increase due BIO		Fold increase due FLOW	
ST flow	221,47			11,52***	RST	159,69			11,76***	RST
Flow	288,45	1,30	TSTT	9,66***	RST	970,99	6,08***	TSTT	6,45***	TSTT
ST static	19,23					13,58				
Static	29,87	1,55*	RST			150,45	11,08***	RST		

In the NRM flow column samples, the corrosion products consisted predominantly of siderite (FeCO₃; 59 vol%), together with appreciable amounts of trivalent iron phases, including goethite (α-FeOOH; 30 vol%) and hematite (α-Fe₂O₃; 11 vol%). Clear differences in corrosion product morphology were also observed between static and flowing conditions (Fig. 3). Under flow conditions, the corrosion products formed a more homogeneous surface layer. After removal of the corrosion products, the underlying steel surface showed no evidence of localized corrosion, in contrast to the static NRM batch experiments.

In contrast, the R2A medium samples showed less dramatic corrosion, although the relative microbial abundances were comparable to the NRM media samples (Fig. 9). Dynamic sterile samples (abiotic) had a mean corrosion loss of 222 g·m⁻²·a⁻¹ (28 μm·a^− 1^), compared to 19 g·m⁻²·a⁻¹ (2 μm·a^− 1^) in static abiotic samples, which equals an 11.5-fold increase and significant difference (Wilcoxon rank sum test, *p* = 9.372 × 10⁻⁷). Inoculated dynamic samples exhibited a mean corrosion loss of 289 g·m⁻²·a⁻¹ (37 μm·a^− 1^), compared to 30 g·m⁻²·a⁻¹ (4 μm·a^− 1^), in static inoculated samples (biotic), representing a 9.7-fold increase due to flow, the difference was statistically significant (Wilcoxon rank sum test, *p* = 4.367 × 10⁻⁶). Under dynamic conditions, the corrosion rates in biotic samples were approximately 1.3 times higher than those in abiotic samples; however, this difference was not statistically significant (Welch’s two-sample t-test, *p* = 0.116). Under static conditions, inoculated samples showed corrosion rates about 1.6 times higher than their sterile counterparts, and the difference was statistically significant (Wilcoxon rank-sum test, *p* = 0.004). The results are summarized in Table 4.

### Microbial composition

DNA extraction from the abiotic control samples yielded concentrations below the Qubit detection threshold, confirming the sterility of these samples. Consequently, the observed corrosion in the sterile controls can be attributed solely to abiotic processes.

PERMANOVA analysis in inoculated samples revealed significant differences in microbial community composition depending on the medium type (NRM vs. R2A; *p* = 0.001), incubation conditions (dynamic (FC) vs. static (B); *p* = 0.001), and sample type (bulk medium vs. swab; *p* = 0.011) (Supplementary Material 2, Supplementary Table S6). Among these factors, incubation conditions accounted for the largest proportion of variability (33%), followed by medium type (30%) and sample type (3%).

**Fig. 10:**
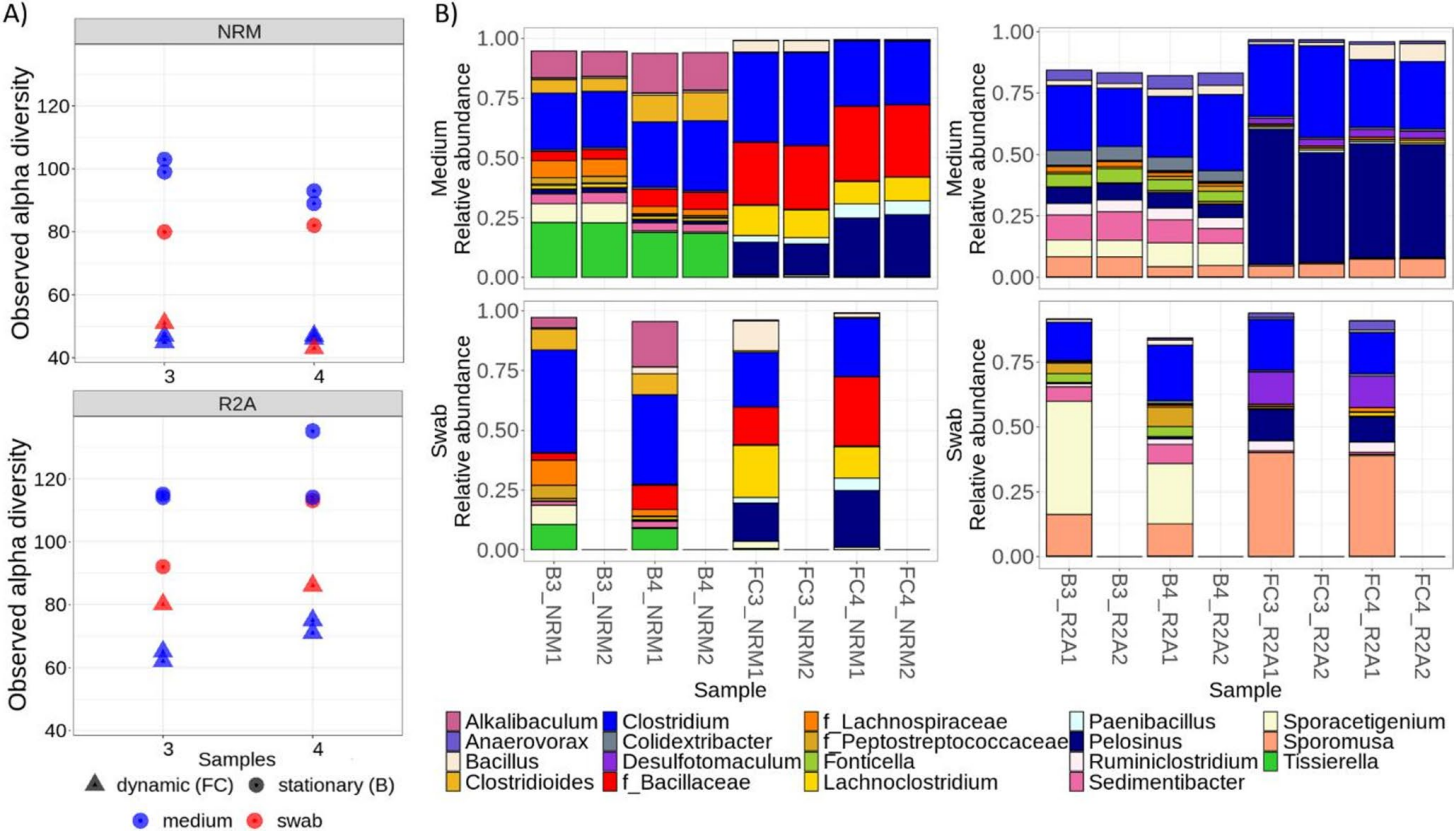
(**A**) Observed alpha diversity in bulk medium and swab samples from flow columns under dynamic (FC) and static (B) conditions. (**B**) Microbial composition detected in the flow column experiment in bulk media and respective swab samples. FC3 and 4 - dynamic flow column samples, B3 and 4 - static column samples. NRM/R2A - Nitrate Broth/R2A medium with 25% of organic content compared to the original prescription; Nr. 1 and 2 - bulk medium DNA extraction replicates (originating from the same flow column), a single swab was analyzed from each column

We further evaluated the observed alpha diversity of samples under dynamic (FC samples) and static conditions (B samples) and the differences between swab and bulk medium samples for both media types. Overall, observed alpha diversity was higher in R2A medium compared to NRM medium. Dynamic conditions consistently reduced alpha diversity relative to static conditions across sample types and media (Fig. 10). In B samples (static conditions), swab samples exhibited lower alpha diversity than the corresponding bulk medium samples for both media. Interestingly, in the case of FC samples (dynamic conditions), alpha diversity in swab and bulk medium samples was comparable for NRM, whereas in R2A medium, swab samples showed even higher alpha diversity than the corresponding bulk media samples (Fig. 10).

To identify microorganisms potentially involved in MIC, we, similar to the Batch experiment, compared the microbial composition of bulk media and swabs in both NRM and R2A media. In the NRM medium, there was no statistically significant difference in microbial composition between the bulk medium and swab (ANOVA, *p* = 0.859). In the dynamic (FC) samples, both bulk medium and swab samples were consistently dominated by *Clostridium*, *Pelosinus*, and members of the Bacillaceae family, with additional contributions from *Lachnoclostridium* and *Paenibacillus*. In contrast, static (B) bulk medium and swab samples showed high abundances of *Clostridium*, *Tissierella*, *Alkalibaculum*, and *Sporacetigenium*, Fig. 10. Detailed analysis of relative differences (%) from the mean abundances of dominant bacterial genera (≥ 5% mean relative frequency) between bulk media and swab samples showed that NRM FC (dynamic) swab samples were specifically enriched in *Lachnoclostridium* and *Bacillus*, whereas NRM B (static) swab samples were enriched in *Clostridium*, *Clostridioides*, as well as members of the families *Lachnospirillaceae* and *Bacillaceae*, Supplementary Material 1, Supplementary Figure S7.

In the R2A medium, a weakly significant difference in microbial composition between bulk medium and swab samples was observed (ANOVA, *p* = 0.024). FC bulk media samples were dominated by *Pelosinus* and *Clostridium*, followed by *Sporomusa*, *Desulfotomaculum*, and *Bacillus*, while FC swab samples were dominated by *Sporomusa*, *Clostridium*, *Desulfotomaculum*, and *Pelosinus*. Similarly, the R2A static (B) bulk media samples were dominated by *Clostridium* together with various less abundant genera such as *Sporacetigenium*, *Sedimentibacter*, *Pelosinus*, *Colidextribacter*, or *Anaerovorax*, while B swab samples were dominated by *Sporoacetigenium*, *Sporomusa*, and *Clostridium*, Fig. 10. Detailed analysis of relative differences (%) from the mean abundances of dominant bacterial genera (≥ 5% mean relative frequency) between bulk media and swab samples showed, that R2A FC (dynamic) swab samples were specifically enriched in *Sporomusa* and *Desulfotomaculum*, while R2A B (static) swab samples exhibited enrichment in *Sporacetigenium* and *Sporomusa* (Supplementary Material 1, Supplementary Figure S7).

## Discussion

This study represents the first phase of a three-stage project investigating microbiologically influenced corrosion (MIC) in Czech bentonite (BCV) relevant to deep geological repositories (DGRs). To balance experimental feasibility with environmental relevance, this phase focuses on mechanistic understanding and process identification rather than direct replication of repository conditions. The objective was to rapidly screen native BCV microorganisms to identify the most corrosive conditions and microbial communities. Experimental conditions were intentionally optimized to promote microbial activity, and simplified batch experiments were used to efficiently compare a wide range of scenarios. The resulting corrosion rates therefore represent conservative, upper-bound estimates of MIC potential. These results provide a screening-level benchmark that informs subsequent project stages and supports conservative assessment of MIC risks and design margins.

### Influence of cultivation conditions and organic carbon availability on MIC in bentonite

Bentonite is a complex matrix hosting diverse indigenous microorganisms, and this study aimed to assess their potential to induce microbiologically influenced corrosion (MIC) under a range of cultivation conditions. Experimental parameters—including medium composition, organic carbon availability, and static versus flow regimes—were systematically varied to capture broad microbial diversity while enriching functionally distinct consortia. In contrast to MIC studies based on pure cultures e.g. (An et al. 2020, 2021; Enning And Garrelfs 2014), this work focused on complex bentonite-derived communities, which more closely reflect natural systems but require interpretation at the level of cultivation conditions rather than individual taxa.

Organic amendments (100% and 25%) were applied to define boundary corrosion conditions and to generate sufficient biomass for reproducible molecular and surface analyses, rather than to replicate repository porewater chemistry. At the same time, zero-organic treatments were included to approximate the low-organic environment expected in DGR bentonite porewaters and to test conditions in which metallic iron may represent the dominant electron donor. Natural organic matter in bentonite is typically present but limited: total organic carbon (TOC) contents reported for bentonites generally range from ~ 0.1 to 0.4 wt% (Marshall et al. 2015), and a TOC of approximately 0.08 wt% has been reported specifically for BCV bentonite (Červinka et al. 2018). The composition and availability of this organic matter may influence microbial reactivation and early-stage activity, particularly during transient phases such as resaturation or redox perturbations (Haynes et al. 2021).

In the present experiments, higher organic content generally promoted greater microbial abundance and metabolically diverse communities, including fermenters and SRB, which are known contributors to MIC. These findings reflect the complex and diverse microbiota associated with BCV bentonite, in agreement with previous bentonite studies (Bartak et al. 2024; Engel et al. 2023; Martinez-Moreno et al. 2024).

However, corrosion rates did not scale directly with biomass: the highest average corrosion rates were generally observed in media without added organic carbon (0%) (Fig. 2; Supplementary material 1, Supplementary Figure S1). This observation is consistent with broader evidence that steel can stimulate or sustain microbial activity in nutrient-poor anoxic subsurface environments, and that corrosion-relevant metabolisms can operate at low cell densities (Rajala et al. 2015). Importantly, corrosion in the 0% organic systems remained predominantly uniform, and no localized (pitting) attack was observed. Because localized corrosion represents a greater threat to canister integrity than uniform metal loss (King et al. 2024), and was therefore a key focus of this study, conditions promoting localized attack were prioritized in subsequent experiments. At the same time, zero-organic media supported sparse and inconsistent microbial populations, and biomass levels were insufficient for robust community characterization or surface-associated analyses, which limited mechanistic interpretation. This constraint reduced the relevance of strictly zero-organic conditions for subsequent experimental stages focused on localized corrosion under flow conditions and in bentonite systems, although selected zero-organic treatments were retained where technically feasible.

Media with reduced organic content (25%) thus represented a critical intermediate condition: sufficiently low to maintain organic limitation and favor corrosion-relevant strategies when readily metabolizable donors are scarce, yet high enough to support analyzable biomass and reproducible surface colonization. This compromise is also defensible from a DGR perspective, where dissolved organic carbon in bentonite porewater is limited but not absent, and where transient conditions—such as early resaturation, localized perturbations, or evolving redox gradients—may temporarily enhance microbial activity and corrosion susceptibility. Under these reduced-organic conditions, enhanced corrosion and, in specific media, localized attack were observed, providing a conservative yet mechanistically informative basis for selecting environments for subsequent experiments.

### Methanogen cultivation limitations

The applied cultivation conditions proved suitable for most targeted microbial groups, with the notable exception of methanogens, which we were unable to cultivate. Although the culture conditions supported the growth of heterotrophic acetogens, aceticlastic methanogens were not detected (Fig. 7). Acetoclastic methanogens, such as *Methanosarcina*, which was detected in long-term anaerobically incubated BCV bentonite suspensions before (KC unpublished data) and which we targeted by choice of the Medium 120 (Koblitz et al. 2023) as a potential corrosive taxon capable of direct electron transfer (Gao And Lu 2021; Holmes et al. 2018), require a strictly anaerobic and reductive environment with low hydrogen partial pressure to thrive (Harris And Schuerger 2025). In cultures dominated by fermentative bacteria and acetogens, as observed in our case, the accumulation of metabolic byproducts (e.g., hydrogen or organic acids) can inhibit the growth of aceticlastic methanogenesis (Kotsyurbenko et al. 2001; Schmidt et al. 2016). In addition, previous studies have reported differing temperature optima for mixed cultures of methanogens and acetogens, with mesophilic conditions (temperature around 30 °C) favoring methanogenesis over acetogenesis (Fu et al. 2019). Since our experiments were conducted at a single temperature (26 °C), it is possible that the chosen temperature conditions were not optimal for promoting methanogens over acetogens. Furthermore, the experimental setup was also not conducive to the growth of hydrogenotrophic methanogens, as we did not supplement the gas phase with H₂ and CO₂, key substrates required for their metabolism (Hanišáková et al. 2022). Effective cultivation would therefore require strictly methanogenic conditions, including incubation under a H₂/CO₂ atmosphere and appropriate redox control, which were beyond the scope of the present experimental design. Further culture experiments are thus needed to define optimal growth conditions for identifying indigenous methanogens in bentonite and estimate their role in MIC.

### Effect of culture transfer on microbial diversity and MIC potential

To select for MIC-capable taxa, we deliberately transferred a small volume (1 mL) of bulk medium from Batch Experiment 1 as inoculum for Batch Experiment 2. The observed alpha diversity patterns in bulk media showed a decrease from Experiment 1 to Experiment 2 across most media types, though the magnitude of this loss varied among media (Fig. 5). High initial alpha diversity followed by a pronounced decrease in Experiment 2 was observed in all R2A samples as well as in NRM25 and PGM25. A consistent, though smaller, decrease occurred across all LB samples. In contrast, negligible diversity loss was observed in NRM100 and PGM100, where alpha diversity was already relatively low in Experiment 1, indicating that selection for dominant taxa had already occurred. This is consistent with previous findings that nutrient-rich conditions often favor rapid proliferation of a few opportunistic species, leading to reduced community alpha diversity (Fierer et al. 2007; Medina et al. 2017). Interestingly, ME medium maintained relatively high and stable alpha diversity across both experiments, regardless of organic content, which implies that it successfully selects for a stable microbial community.

Although corrosion rates were not significantly higher compared to sterile controls, likely due to the limited number of corrosion coupons and high variability, the highest rates in NRM, PGM, and R2A media were recorded during Experiment 2. Similarly, the largest relative corrosion losses (compared to abiotic controls) were observed also in Experiment 2 (Fig. 2). These results indicate successful enrichment of more aggressive taxa in Experiment 2.

Importantly, only the bulk medium (i.e., planktonic fraction) was transferred, while biofilm material was not. As a result, biofilm-associated taxa may have been lost during the transfer. Co-transfer of biofilm with the bulk medium was not feasible due to the limited number of coupons, and we prioritized collecting biofilm samples via swabbing to obtain compositional data rather than using them for the transfer. However, alpha diversity in Experiment 1 was comparable between bulk and swab samples for most media, with bulk samples showing slightly higher alpha diversity in all the samples except PGM, where the patterns were probably negatively influenced by the technical difficulties of NGS in bulk media samples. Swab samples also exhibited a smaller alpha diversity decrease between experiments than bulk media across all the media (Fig. 5), implying that the transfer did not result in a major loss of biofilm-associated alpha diversity. Moreover, dominant taxa were consistently detected in both bulk and swab samples in Experiment 1, suggesting that the main functional members of the community were retained. Together with the finding that 25% organic content media maintained higher alpha diversity, these observations support the use of reduced-organic-content media for the planned flow cell experiments. A similar approach, using bentonite pre-culture as inoculum, was applied in the subsequent flow column study.

### Microbial composition in bulk medium and swab biofilms – Identification of MIC taxa

When working with complex microbial communities and aiming to infer the ecological roles of specific taxa, such as identifying those responsible for MIC, one of the most straightforward approaches is to compare the microbial composition of the bulk medium with that found directly on the surface of metal coupons. Studies have shown that microbial communities forming biofilms on metal surfaces often differ substantially in composition and function from those in the surrounding planktonic phase (Dang And Lovell 2000). This distinction is particularly important in the context of MIC, as electrochemical MIC (EMIC) typically requires direct contact between microorganisms and the metal surface, promoting biofilm formation (Enning And Garrelfs 2014). In contrast, chemically mediated MIC (CMIC) can occur without direct contact between microbial cells and the metal surface; as such, it may be driven not only by surface-attached biofilms but also by planktonic cells that release corrosive metabolites—such as protons, organic acids, or sulfur species—into the surrounding environment (Knisz et al. 2023). A more advanced strategy involves macroecological approaches, such as identifying taxa that consistently co-occur with corrosion-related features (e.g., elevated corrosion rates or visible corrosion damage in our case). One such high-tech method is the Data-driven Keystone species Identification (DKI) framework, which employs deep learning to detect taxa that play pivotal roles in microbial community dynamics (Wang et al. 2024). These methods can help pinpoint potential keystone species associated with corrosion-prone environments. However, co-occurrence network analysis and DKI require a large number of samples to enable robust statistical comparisons and a detailed understanding of the ecological factors shaping microbial composition. In our study, the number of replicates was limited, and the variability in environmental parameters and corrosion data was too high to apply these advanced approaches effectively. Performed correlation analysis linking microbial diversity with elevated corrosion rates across Batch experiments did not yield any statistically significant taxa. Therefore, we focused on identifying taxa that were specifically enriched in swab samples taken from the coupon surfaces in experiments where the highest corrosion rates were observed. This targeted approach allowed us to narrow down potential MIC-relevant taxa despite the limitations in sample size and variability.

### Bentonite taxa associated with MIC

Microbial communities in Batch experiments and Flow column experiments shared some dominant genera such as *Clostridium* or *Pelosinus* (Figs. 6, 7 and 10). However, swabbed biofilms from samples exhibiting elevated corrosion rates in both experimental setups showed a marked enrichment of specific genera, including *Clostridium*, *Clostridioides*, *Bacillus*, *Lachnoclostridium*, *Sporomusa*, *Sporoacetigenium*, *Desulfofarcimen*, *Desulfovectis*, *Desulfovibrio*, and *Desulfotomaculum*. These taxa have been identified as potential MIC-causing microorganisms present in Czech BCV bentonite.

The corrosion rate estimated in Batch experiment under static conditions was 11 μm·a⁻¹ in sterile pore water. This rate is typical during the initial months of exposure to Czech bentonite, as reported by (Forman et al. 2021). In contrast, in the NRM medium, we observed an increase in the overall static corrosion rate to nearly 60 μm·a⁻¹, and the corrosion was strongly localized, reaching peak local corrosion penetration rate up to 160 μm·a⁻¹. These results led to the selection of this cultivation condition for the flow column experiment, as well as for the subsequent electrochemical measurements performed in stage 2 of our project. The coupon-surface-attached (swab) communities in these highly-corroded samples were dominated by *Clostridium* and/or *Clostridioides* spp. (Fig. 6). *Clostridium* and *Clostridioides* spp. are fermentative bacteria capable of producing acetate, organic acids, and hydrogen, with some strains also exhibiting sulfate-reducing activity (Bergey et al. 2015). Although we unfortunately have not measured pH in our samples, these metabolic processes are suspected to enhance corrosion by lowering the environmental pH and providing electron donors (e.g. acetate or hydrogen) for other microorganisms—a mechanism described by (Akvan And Javaherdashti 2019). In addition, these species can also contribute to biofilm formation, creating localized microenvironments conducive to corrosion. For example, (Xu et al. 2024) reported a comparable corrosion rate of 60 μm·a⁻¹ after just seven days of exposure to *Clostridioides difficile*, accompanied by pitting corrosion on martensitic stainless steel AISI420, with extreme local corrosion rates reaching 213 μm·a⁻¹. They also demonstrated the electrochemical activity of *Clostridioides* biofilms. Similarly, significant corrosion (110 μm·a⁻¹) was also observed for *Clostridium bifermentans*, which induced localized corrosion with pit diameters up to 50 μm (Palaniappan And Toleti 2016). The pit observed in our sample NRM25 in Batch experiment 2, with the highest corrosion rate (60 μm·a⁻¹), measured 600 μm in diameter and had a depth of about 40 μm after 3 months of exposure (Fig. 4). The electroactivity in *Clostridium* has been independently confirmed by other studies (Schwab et al. 2019; Zani et al. 2024), although a direct link to corrosion has not always been established. Interestingly, while *Clostridium* species were dominant in many potentially corrosion-associated microbial communities in our experiments, their presence did not consistently correlate with high corrosion rates. Several samples showed low corrosion rates despite high relative abundances of *Clostridium* (Figs. 6 and 7), suggesting that their mere presence is not a reliable indicator of MIC. This observation indicates that *Clostridium* likely contributes to corrosion but in a context-dependent manner, influenced by both environmental conditions and the surrounding microbial community.

In addition to *Clostridium*, other fermentative and acetogenic taxa were identified on corrosion coupon surfaces associated with elevated corrosion rates. In dynamic NRM samples, which exhibited the highest overall corrosion rates but no signs of localization, *Lachnoclostridium* and *Bacillus* were dominant. Furthermore, in R2A samples, we detected other acetogenic taxa such as *Sporomusa* or *Sporoacetogenium*. While there are no specific reports on MIC or electrochemical activity in *Lachnoclostridium*, its close phylogenetic relationship to *Clostridium* suggests it may share similar properties. Likewise, no particular information is available for *Sporoacetogenium*. The genus *Sporomusa*, however, has been shown to directly utilize electrons from iron under anoxic, sulfate-free, freshwater conditions, contributing to corrosion through acetate production. Still, only a limited number of acetogenic species possess mechanisms for efficiently harnessing cathodic electrons from Fe(0) oxidation (Kato et al. 2015).

The genus *Bacillus* and other representatives of the Bacillaceae family belong to heterotrophic nitrate-reducing bacteria, which were frequently detected in swab samples with elevated corrosion rates across several experimental conditions, most notably in NRM dynamic flow column experiments. *Bacillus* species and various other bacilli were also found in samples with media lacking organic carbon, where elevated corrosion rates were still observed.

The impact of *Bacillus* on steel corrosion is notably strain-specific: certain strains accelerate degradation, while others appear to confer protection through robust biofilm formation. Several studies demonstrate that *Bacillus* species, particularly nitrate-reducing strains, can markedly impact steel corrosion under different environmental contexts. For example, *B. cereus* biofilms in nitrate-rich soil accelerated pitting on both X80 pipeline steel and 304 stainless steel, with corrosion severity modulated by biofilm state and oxygen levels (Liu et al. 2021). Similarly, *B. cereus* significantly promoted pitting corrosion, depths reaching approximately 11.23 μm after 60 days of exposure on X80 pipeline steel under soil conditions (Wan et al. 2018). In contrast, biofilms formed by *Bacillus* sp. Y6 effectively reduced corrosion on Q235 carbon steel (Ruan et al. 2023). Specific environmental conditions like nitrate availability, nutrient content, and oxygen levels were found to substantially influence corrosion outcomes across multiple studies, e.g., (Huang et al. 2021; Salgar-Chaparro et al. 2020). Another heterotrophic genus detected in our samples was *Alkalibacter*, which was identified specifically in NRM samples, but no MIC-specific information could be found.

The last potentially harmful group identified in our samples is sulfate-reducing bacteria (SRB). Genera such as *Desulfofarcimen*, *Desulfovectis*, *Desulfovibrio*, or *Desulfotomaculum* were enriched explicitly on the surfaces of corrosion coupons with elevated corrosion rates in PGM and R2A media, indicating their potential role in microbiologically influenced corrosion. SRB are the most consistently implicated microbial group in MIC within DGR research. Their activity can induce localized pitting and lead to the formation of sulfide-containing corrosion products such as FeS or mackinawite (Necib et al. 2017). Except for CMIC, some of the MIC-inducible SRB strains, such as *Desulfovibrio ferrophilus* or *Desulfopila* corrodens, are also capable of withdrawing electrons directly from Fe(0) rather than consuming abiotically generated H2, although the molecular mechanisms remain unknown (Enning et al. 2012; Enning And Garrelfs 2014). *Desulfovibrio desulfuricans* is a frequently studied SRB causing MIC. Corrosion rates for *D. desulfuricans* have been reported to reach 1.2 μm·a⁻¹ in water and 0.5 μm·a⁻¹ in clay environments and were higher in the presence of Postgate B medium compared to the medium without it (Sattar et al. 2024). Similarly, *Desulfovibrio* together with *Desulfomicrobium* were identified as MIC-causing bacteria by (Černoušek et al. 2020). In their study, the average corrosion rate was 13 μm·a⁻¹ for the sterile sample and 48 μm·a⁻¹ for the non-sterile sample. The maximum localized penetration rate reached 22 μm·a⁻¹ in the sterile sample and 106 μm·a⁻¹ in the non-sterile sample, while the localization factor increased only slightly upon inoculation, from 1.7 to 2.2. *Desulfotomaculum* species, including *D. nigrificans* and *D. geothermicum*, have also demonstrated considerable corrosive potential. For instance, D. nigrificans caused a sixfold increase in weight loss compared to controls, while *D. geothermicum*, a thermophilic strain, induced corrosion at a rate of 70 μm·a⁻¹ at 50 °C (Anandkumar et al. 2009; Liu et al. 2019). Despite the extensive research on *Desulfovibrio* and *Desulfotomaculum* species, no studies were found on the genus *Desulfofarcimen* or *Desulfovectis* identified in our samples, indicating a gap in the current understanding of their potential role in MIC.

### Dynamic flow effects – Implications for DGR

Dynamic flow conditions in deep geological repositories (DGRs) are considered highly unlikely under planned operational scenarios. Bentonite swelling is expected to rapidly seal any technological gaps that could result in advective water flow (Hausmannová et al. 2023; Svoboda et al. 2022). Although chemical erosion via colloid formation is improbable in Czech bivalent bentonites, safety assessments must also account for extreme or transient scenarios that simulate hydraulic disturbances to test system robustness (Pont et al. 2020; Sellin 2017), such as preferential pathways formed by uneven saturation or gas breakthrough (Marschall et al. 2024). For these reasons, our flow column experiments were designed to represent such conservative worst-case conditions and test their effect on corrosion processes. The continuous flow maintains a steady nutrient supply and removes corrosion products, thereby enhancing metabolite transport, biofilm turnover, and redox gradients (An et al. 2020; Knisz et al. 2023). This setup therefore accelerated the assessment of MIC and helped reveal underlying mechanisms that might otherwise remain undetected within the experimental time frames under diffusion-limited conditions.

Our results demonstrated that dynamic conditions significantly increased corrosion rates compared to static systems, both in abiotic controls and in inoculated samples. In our dynamic flow column experiment with NRM medium, we observed that dynamic flow sterile samples exhibited corrosion rates nearly twelve times higher than those under static conditions, indicating a substantial abiotic contribution to corrosion (Fig. 9). This indicates that dynamic flow alone, even in the absence of microbial activity, can enhance corrosion. Several studies have shown that fluid shear stress under dynamic conditions can accelerate corrosion by increasing the interaction between corrosive agents and metal surfaces and by disrupting protective corrosion product layers, thereby reducing their effectiveness (Gao et al. 2024; Liu et al. 2022).

In addition to these abiotic effects, dynamic conditions can further amplify the impact of microbial corrosion. In the NRM medium, the corrosion rate in inoculated dynamic flow samples was approximately six times higher than in sterile dynamic flow samples (Fig. 9). The combined influence of biotic and abiotic factors led to a total corrosion rate increase of up to 72-fold in inoculated dynamic flow samples compared to sterile controls. This highlights the strong synergistic impact of both dynamic flows replenishing the nutrients and removing the inhibitory byproducts and microbial activity on corrosion acceleration. This observation aligns with findings from An et al. (2020), who reported markedly higher corrosion rates in dynamic systems compared to diffusion-limited environments.

Additional insight into the influence of flow conditions was provided by analysis of corrosion product composition and morphology in the NRM flow column experiments. In contrast to static systems, corrosion products formed under dynamic flow conditions included not only siderite but also appreciable proportions of trivalent iron phases. Such phases are uncommon under strictly anoxic conditions, but have previously been reported in nitrate-containing systems (Shrestha et al. 2021). Their occurrence may indicate a higher oxidizing capacity in the flowing NRM system, potentially associated with nitrate availability and enhanced mass transport, although direct evidence for the underlying mechanisms was not obtained in this study. Morphological differences were also observed: under dynamic flow, corrosion products formed a more homogeneous surface layer, and after their removal, the underlying steel surface showed no signs of localized attack, in contrast to static NRM batch and static flow column experiments (Fig. 3).

Consistent with these observations, no localized corrosion was detected on coupons exposed to dynamic flow in the NRM medium, despite substantially elevated corrosion rates. This contrasts with NRM Batch Experiment 2 and static flow column conditions, where localized attack was observed (Figs. 3 and 4) and NRM static flow column samples. Notably, the microbial composition also differed between these two conditions: corrosion coupon swabs from dynamic flow column NRM samples were enriched in *Lachnoclostridium* and *Bacillus* (Fig. 10), whereas the Batch experiment 2 swab samples were predominantly enriched in *Clostridium*/*Clostridioides* (Fig. 6), similarly to the static NRM samples in the flow column experiment (Fig. 10). This shift in microbial community structure is likely influenced by the continuous removal of corrosion products under flow conditions, which can prevent the formation of aggressive microenvironments necessary for localized corrosion and can also drive changes in biofilm composition and structure. Our results imply that static conditions promote heterogeneous biofilm development, leading to localized corrosion. This interpretation is supported by the measured maximal localized penetration corrosion rates 160 μm·a⁻¹ under static conditions, indicating localized corrosion, versus a more uniform rate of 124 μm·a⁻¹ under dynamic flow (Table 4).

In contrast, the R2A medium showed high corrosion rate differences between static and dynamic samples due to flow, but only a mild increase in the corrosion rates of inoculated samples compared to sterile controls under both static and dynamic conditions (Fig. 9), although the detected relative microbial abundances were even higher in FC R2A samples than in FR NRM samples (Fig. 9). This implies a relatively weak effect of biological factors on corrosion in this medium. Also, no localized corrosion was observed in R2A medium samples under either static or flow conditions, suggesting that microbial communities present in R2A exerted a less aggressive or more uniformly distributed corrosive effect.

These findings underscore the critical importance of considering both abiotic and biotic factors in corrosion risk assessments for DGR. Although dynamic flow conditions represent an extreme scenario for DGR, our data clearly demonstrate that even localized water movement or minor breaches could create microenvironments highly conducive to MIC. The synergistic interaction between fluid dynamics and microbial activity can result in a substantial increase in corrosion rates, posing a significant risk to the long-term integrity and safety of repository barriers.

### Microbiologically induced corrosion inhibition (MICI)

In most media types tested, corrosion increased following inoculation with bentonite-derived microorganisms. However, the ME medium was an exception, where sterile controls exhibited higher corrosion rates than the inoculated samples (with one exception). This observation suggests that abiotic factors, particularly the chemical composition of the medium, can significantly influence corrosion processes and, in some cases, dramatically enhance corrosion even in the absence of microbial activity. The elevated corrosion rates observed in sterile ME medium (up to 47 μm·a⁻¹) may be related to its relatively high chloride content (Table 1). Although LB medium contains an even higher chloride concentration, free amino acids derived from tryptone present in this media, are known to act as corrosion inhibitors (El-Sayed 2016; Messina et al. 2023), which may explain the lower corrosion rates observed in LB compared to ME.

On the other hand, the corrosion rates in inoculated ME medium are in most set-ups significantly lower than in sterile samples (Fig. 2), suggesting that microbial surface colonization may have mitigated corrosion under these conditions. Such behavior is consistent with microbiologically influenced corrosion inhibition (MICI), a phenomenon in which biofilm formation reduces corrosion rates. MICI has been reported to depend on the presence of an intact, living biofilm, with corrosion resuming once the biofilm is disrupted, for example by antibiotic treatment(Scardino et al. 2025). A recent review by Li et al. (Li et al. 2025) summarized the diverse mechanisms, such as oxygen depletion, secretion of antimicrobial compounds, competitive exclusion of corrosive species or biomineralization, by which microorganisms can mitigate metal corrosion.

In the ME medium, a markedly elevated corrosion rate was observed in only one sample (Fig. 7). Although the corresponding swab sample was dominated by Paraclostridium, this taxon was also abundant in other ME100 samples that exhibited much lower corrosion rates, and no consistent microbial pattern was associated with either high- or low-corrosion coupons. For example, the replicate sample ME100_B, prepared under identical conditions, displayed a different community composition yet similarly low corrosion. These observations suggest that corrosion inhibition in inoculated ME systems is more likely related to biofilm presence and integrity than to specific microbial taxa, and that localized biofilm disruption, potentially occurring in ME100_A, may have allowed corrosion to proceed. Further targeted experiments are required to evaluate the role of bentonite-associated microorganisms in microbiologically influenced corrosion inhibition (MICI) under ME conditions.

### Relevance of results for subsequent project stages

This study constituted the initial stage of a multi-stage investigation of microbiologically influenced corrosion (MIC) in Czech bentonite (BCV) under controlled experimental conditions relevant to deep geological repositories (DGRs). Given the need to balance environmental realism with experimental feasibility, this stage focused on mechanistic screening and process identification rather than direct replication of repository conditions. Consequently, the results provide conservative, upper-bound estimates of MIC potential rather than predictive in situ corrosion rates.

The primary objective was to identify microbial consortia and environmental conditions most relevant for carbon steel corrosion in bentonite-based barrier systems. Screening experiments under static and dynamic conditions showed that nitrate-containing media (NRM) and reduced-organic heterotrophic media (particularly R2A and selected PGM variants) consistently promoted enhanced microbial activity and elevated corrosion rates. Localized corrosion was observed exclusively in NRM, whereas R2A and PGM supported increased but predominantly uniform corrosion. These systems were therefore identified as representative high-risk environments for further investigation.

Subsequent project work built directly on these findings. Electrochemical experiments targeted MIC mechanisms under the most aggressive conditions identified, particularly in NRM-based systems, to assess the role of electrochemically mediated microbial activity. In parallel, the screening results were translated into bentonite-containing systems, progressing from suspensions to compacted bentonite to evaluate the influence of bentonite presence, confinement, and restricted transport on microbial activity and corrosion behavior.

Overall, this stepwise approach ensured that subsequent experiments were grounded in experimentally identified worst-case scenarios. By systematically transferring the most aggressive microbial systems into increasingly constrained environments, the project maintained a conservative framework while enhancing the relevance of the results for MIC risk assessment, canister design, and safety margins in DGR concepts.

## Conclusion

This study demonstrates that bentonite-associated microbial communities can significantly influence corrosion processes under experimentally controlled conditions that capture selected processes relevant to deep geological repositories (DGRs). The most aggressive conditions were identified in NRM culture medium, where localized corrosion and the formation of oxidized corrosion products such as goethite and hematite were observed. These features, together with the presence of biofilm detected within corrosion pits, suggest an increased redox potential and accelerated cathodic reactions, likely linked to the coupling of metal oxidation with microbial nitrate reduction. Across different cultivation conditions, elevated corrosion was primarily associated with fermentative, acetogenic heterotrophic, and SRB taxa such as *Clostridium*, *Clostridioides*, *Bacillus*, *Lachnoclostridium*, *Sporomusa*, *Sporoacetigenium*, *Desulfofarcimen*, *Desulfovectis*, *Desulfovibrio*, and *Desulfotomaculum*. A pronounced synergistic effect between microbial activity and dynamic flow was observed, increasing corrosion rates by up to 72-fold under nutrient-replenished, flow-through conditions.

The enrichment of diverse anaerobic taxa and consistently elevated corrosion rates across different incubation conditions demonstrate that bentonite harbors metabolically active consortia capable of driving steel corrosion in DGR-like settings. Distinct microbial communities detected on metal surfaces, compared to bulk cultures, highlight the need to focus future MIC research on surface-associated biofilms, where synergistic metabolic interactions and electron transfer processes are likely key drivers of corrosion. The results further indicate that biofilm formation plays an important role in mediating MIC in organic-rich environments, where localized disruption of biofilm integrity may further accelerate corrosion.

This work represents the first phase of a structured, three-stage investigation designed to address MIC mechanisms under increasing system complexity. The second phase focuses on mechanistic electrochemical studies of electron transfer and localized corrosion under the most aggressive conditions in NRM media identified here, while the third involves long-term corrosion experiments in compacted, saturated bentonite under near-repository conditions. The outcomes of these subsequent stages will be published separately. Together, the three phases form a coherent research framework to refine corrosion rate estimates and strengthen the reliability of safety assessments for DGR systems—an essential element in national strategies for the safe, long-term management of nuclear waste. This is particularly critical as nuclear energy is increasingly recognized as a key contributor to low-carbon energy transitions.

## Supplementary Information

Below is the link to the electronic supplementary material.

Supplementary Material 1 (DOCX 63.8 KB)

Supplementary Material 2 (DOCX 3.92 MB)

## Data Availability

Sequencing data: NCBI BioProject PRJNA1311298. Other data are in the manuscript, supplementary files, or available from the corresponding author upon request.

## Declarations

### Generative AI and AI-assisted technologies in the writing process

During the preparation of this work, the author(s) used Microsoft CoPilot and OpenAI ChatGTP 5.0 in order to improve the formulations and clarity of the text. After using this service, the author(s) reviewed and edited the content as needed and take(s) full responsibility for the content of the publication.

## References

[CR1] Agency OECDNE (2003) Engineered barrier systems and the safety of deep geological repositories: state-of-the-art report, radioactive waste management. OECD. 10.1787/9789264103450-en

[CR2] Ahmed MA, Mahmoud SA, Mohamed AA (2024) Unveiling the complexities of microbiologically induced corrosion: mechanisms, detection techniques, and mitigation strategies. Front Environ Sci Eng 18:120. 10.1007/s11783-024-1880-8

[CR3] Akvan F, Javaherdashti R (2019) Failure modes, effects and causes of microbiologically influenced corrosion

[CR6] Anandkumar B, Rajasekar A, Venkatachari G, Maruthamuthu S (2009) Effect of thermophilic sulphate-reducing bacteria (Desulfotomaculum geothermicum) isolated from Indian petroleum refinery on the corrosion of mild steel. Curr Sci 97

[CR5] An BA, Deland E, Sobol O, Yao J, Skovhus TL, Koerdt A (2021) The differences in the corrosion product compositions of Methanogen-induced microbiologically influenced corrosion (Mi-MIC) between static and dynamic growth conditions. Corros Sci 180:109179. 10.1016/j.corsci.2020.109179

[CR4] An BA, Kleinbub S, Ozcan O, Koerdt A (2020) Iron to gas: versatile multiport flow-column revealed extremely high corrosion potential by methanogen-induced microbiologically influenced corrosion (Mi-MIC). Front Microbiol 11 10.3389/fmicb.2020.00527 PMC7136402 32296410

[CR8] Bartak D, Šachlová Š, Kašpar V, Říha J, Dobrev D, Večerník P, Hlaváčková V, Matulová M, Černá K (2024) Dramatic loss of microbial viability in bentonite exposed to heat and gamma radiation: implications for deep geological repository. World J Microbiol Biotechnol 40:264. 10.1007/s11274-024-04069-w 38990244 10.1007/s11274-024-04069-w PMC11239606

[CR7] Bartak D, Bedrníková E, Kašpar V, Říha J, Hlaváčková V, Večerník P, Šachlová Š, Černá K (2023) Survivability and proliferation of microorganisms in bentonite with implication to radioactive waste geological disposal: strong effect of temperature and negligible effect of pressure. World J Microbiol Biotechnol 40:41. 10.1007/s11274-023-03849-0 38071262 10.1007/s11274-023-03849-0 PMC10710388

[CR9] Bartak D, Říha J, Dudáš D, Gallus P, Bedrníková E, Kašpar V, Černá K (2025) Bentonite sterilization methods in relation to geological disposal of radioactive waste: comparative efficiency of dry heat and gamma radiation. J Appl Microbiol lxaf051. 10.1093/jambio/lxaf051 40042982 10.1093/jambio/lxaf051

[CR11] Bengtsson A, Edlund J, Hallbeck B, Heed C, Pedersen K (2015) Microbial sulphide-producing activity in MX-80 bentonite at 1750 and 2000 kg m–3 wet density. (SKB report No. R-15-05). Swedish Nuclear Fuel and Waste Management Co, Stockholm, Sweden

[CR10] Bengtsson A, Pedersen K (2016) Microbial sulphate-reducing activity over load pressure and density in water saturated Boom Clay. Appl Clay Sci 132–133:542–551. 10.1016/j.clay.2016.08.002

[CR12] Bergey DH, Harrison FC, Breed RS, Hammer BW, Huntoon FM (eds) (2015) Bergey’s Manual of Systematics of Archaea and Bacteria. Wiley Online Library

[CR13] Bolyen E, Rideout JR, Dillon MR, Bokulich NA, Abnet CC, Al-Ghalith GA, Alexander H, Alm EJ, Arumugam M, Asnicar F, Bai Y, Bisanz JE, Bittinger K, Brejnrod A, Brislawn CJ, Brown CT, Callahan BJ, Caraballo-Rodríguez AM, Chase J, Cope EK, Da Silva R, Diener C, Dorrestein PC, Douglas GM, Durall DM, Duvallet C, Edwardson CF, Ernst M, Estaki M, Fouquier J, Gauglitz JM, Gibbons SM, Gibson DL, Gonzalez A, Gorlick K, Guo J, Hillmann B, Holmes S, Holste H, Huttenhower C, Huttley GA, Janssen S, Jarmusch AK, Jiang L, Kaehler BD, Kang KB, Keefe CR, Keim P, Kelley ST, Knights D, Koester I, Kosciolek T, Kreps J, Langille MGI, Lee J, Ley R, Liu Y-X, Loftfield E, Lozupone C, Maher M, Marotz C, Martin BD, McDonald D, McIver LJ, Melnik AV, Metcalf JL, Morgan SC, Morton JT, Naimey AT, Navas-Molina JA, Nothias LF, Orchanian SB, Pearson T, Peoples SL, Petras D, Preuss ML, Pruesse E, Rasmussen LB, Rivers A, Robeson MS, Rosenthal P, Segata N, Shaffer M, Shiffer A, Sinha R, Song SJ, Spear JR, Swafford AD, Thompson LR, Torres PJ, Trinh P, Tripathi A, Turnbaugh PJ, Ul-Hasan S, van der Hooft JJJ, Vargas F, Vázquez-Baeza Y, Vogtmann E, von Hippel M, Walters W, Wan Y, Wang M, Warren J, Weber KC, Williamson AD, Xu ZZ, Zaneveld JR, Zhang Y, Zhu Q, Knight R, Caporaso JG (2019) Reproducible, interactive, scalable and extensible microbiome data science using QIIME 2. Nat Biotechnol 37:852–857. 10.1038/s41587-019-0209-9 31341288 10.1038/s41587-019-0209-9 PMC7015180

[CR14] Bonsu DNO, Higgins D, Simon C, Henry JM, Austin JJ (2024) Metal–DNA interactions: Exploring the impact of metal ions on key stages of forensic DNA analysis. Electrophoresis 45:779–793. 10.1002/elps.202300070 37638716 10.1002/elps.202300070

[CR15] Callahan BJ, McMurdie PJ, Rosen MJ, Han AW, Johnson AJA, Holmes SP (2016) DADA2: High resolution sample inference from Illumina amplicon data. Nat Methods 13:581–583. 10.1038/nmeth.3869 27214047 10.1038/nmeth.3869 PMC4927377

[CR16] Černoušek T, Shrestha R, Kovářová H, Špánek R, Ševců A, Sihelská K, Kokinda J, Stoulil J, Steinová J (2020) Microbially influenced corrosion of carbon steel in the presence of anaerobic sulphate-reducing bacteria. Corros Eng Sci Technol 55:127–137. 10.1080/1478422X.2019.1700642

[CR17] Červinka R, Vašíček R, Večerník P, Kašpar V (2018) Kompletní charakterizace bentonitu BCV 2017 (No. SÚRAO TZ 419/2019)

[CR18] Chen J, Qin Z, Shoesmith DW (2011) Long-term corrosion of copper in a dilute anaerobic sulfide solution. Electrochim Acta Electrochem IMPEDANCE Spectrosc 56:7854–7861. 10.1016/j.electacta.2011.04.086

[CR110] Claesson MJ, Wang Q, O’sullivan O, Greene-Diniz R, Cole JR, Ross RP, O'Toole PW (2010) Comparison of two next-generation sequencing technologies for resolving highly complex microbiota composition using tandem variable 16S rRNA gene regions. Nucleic Acids Res 38:e200–e200 20880993 10.1093/nar/gkq873 PMC3001100

[CR19] Clifford RJ, Milillo M, Prestwood J, Quintero R, Zurawski DV, Kwak YI, Waterman PE, Lesho EP, Mc Gann P (2012) Detection of bacterial 16S rRNA and identification of four clinically important bacteria by real-time PCR. PLoS ONE. 10.1371/journal.pone.0048558 23139793 10.1371/journal.pone.0048558 PMC3490953

[CR20] Cornell RM, Schwertmann U (2003) The Iron Oxides: Structure, Properties, Reactions, Occurrences and Uses. Wiley

[CR21] Dang H, Lovell CR (2000) Bacterial Primary Colonization and Early Succession on Surfaces in Marine Waters as Determined by Amplified rRNA Gene Restriction Analysis and Sequence Analysis of 16S rRNA Genes. Appl Environ Microbiol 66:467–475. 10.1128/aem.66.2.467-475.2000 10653705 10.1128/aem.66.2.467-475.2000 PMC91850

[CR22] Davis NM, Proctor DM, Holmes SP, Relman DA, Callahan BJ (2018) Simple statistical identification and removal of contaminant sequences in marker-gene and metagenomics data. bioRxiv. 10.1101/221499 PMC6298009 30558668

[CR109] Dowd SE, Callaway TR, Wolcott RD, Sun Y, McKeehan T, Hagevoort RG, Edrington TS (2008) Evaluation of the bacterial diversity in the feces of cattle using 16S rDNA bacterial tag-encoded FLX amplicon pyrosequencing (bTEFAP). BMC Microbiol 8:125. 10.1186/1471-2180-8-125 18652685 10.1186/1471-2180-8-125 PMC2515157

[CR23] El-Sayed NH (2016) Corrosion inhibition of carbon steel in chloride solutions by some amino acids. Eur J Chem 7:14–18. 10.5155/eurjchem.7.1.14-18.1331

[CR24] Engel K, Ford SE, Binns WJ, Diomidis N, Slater GF, Neufeld JD (2023) Stable microbial community in compacted bentonite after 5 years of exposure to natural granitic groundwater. mSphere 8:e00048–e00023. 10.1128/msphere.00048-23 37772811 10.1128/msphere.00048-23 PMC10597416

[CR25] Enning D, Garrelfs J (2014) Corrosion of iron by sulfate-reducing bacteria: new views of an old problem. Appl Environ Microbiol 80:1226–1236. 10.1128/AEM.02848-13 24317078 10.1128/AEM.02848-13 PMC3911074

[CR26] Enning D, Venzlaff H, Garrelfs J, Dinh HT, Meyer V, Mayrhofer K, Hassel AW, Stratmann M, Widdel F (2012) Marine sulfate-reducing bacteria cause serious corrosion of iron under electroconductive biogenic mineral crust. Environ Microbiol 14:1772–1787 22616633 10.1111/j.1462-2920.2012.02778.x PMC3429863

[CR27] Fierer N, Bradford MA, Jackson RB (2007) Toward an ecological classification of soil bacteria. Ecology 88:1354–1364. 10.1890/05-1839 17601128 10.1890/05-1839

[CR28] Forman L, Picek M, Dobrev D, Gondolli J, Miranda M, Straka M, Kouřil M, Stoulil J, Matal O, Čermák J, Král J, Žaloudek J, Vávra M, Čupr M (2021) Research and development of a disposal package for the deep disposal of spent nuclear fuel to the sample implementation stage (No. Technical report 544/2021). SÚRAO

[CR29] Fu B, Jin X, Conrad R, Liu H, Liu H (2019) Competition between chemolithotrophic acetogenesis and hydrogenotrophic methanogenesis for exogenous H2/CO2 in anaerobically digested sludge: Impact of temperature. Front Microbiol 10:2418. 10.3389/fmicb.2019.02418 31749772 10.3389/fmicb.2019.02418 PMC6842956

[CR30] Gaby JC, Buckley DH (2017) The use of degenerate primers in qPCR analysis of functional genes can cause dramatic quantification bias as revealed by investigation of nifH primer performance. Microb Ecol 74:701–708. 10.1007/s00248-017-0968-0 28389727 10.1007/s00248-017-0968-0

[CR31] Gao K, Lu Y (2021) Putative extracellular electron transfer in methanogenic Archaea. Front Microbiol. 10.3389/fmicb.2021.611739 33828536 10.3389/fmicb.2021.611739 PMC8019784

[CR32] Gao Q, Lu Y, Jia J, Wu Y, Han X, Zhang C (2024) Effect of nitrate-carbonate molten salt flow rate for the 347H corrosion behavior. J Phys Conf Ser 2760:012074. 10.1088/1742-6596/2760/1/012074

[CR33] Gilmour KA, Davie CT, Gray N (2021) An indigenous iron-reducing microbial community from MX80 bentonite - A study in the framework of nuclear waste disposal. Appl Clay Sci 205:106039. 10.1016/j.clay.2021.106039 10.1016/j.scitotenv.2021.152660 34958843

[CR34] Gilmour KA, Davie CT, Gray N (2022) Survival and activity of an indigenous iron-reducing microbial community from MX80 bentonite in high temperature / low water environments with relevance to a proposed method of nuclear waste disposal. Sci Total Environ 814:152660. 10.1016/j.scitotenv.2021.152660 34958843 10.1016/j.scitotenv.2021.152660

[CR35] Hajj HE, Abdelouas A, Grambow B, Martin C, Dion M (2010) Microbial corrosion of P235GH steel under geological conditions. Phys Chem Earth Parts ABC 35:248–253. 10.1016/j.pce.2010.04.007

[CR36] Hanišáková N, Vítězová M, Rittmann S-M (2022) The historical development of cultivation techniques for methanogens and other strict anaerobes and their application in modern microbiology. Microorganisms 10:412. 10.3390/microorganisms10020412 35208865 10.3390/microorganisms10020412 PMC8879435

[CR37] Harper CO, Brown JL, Amos RT (2024) Corrosion processes affecting copper-coated used fuel containers for the disposal of spent nuclear fuel: critical review of the state-of-knowledge. Npj Mater Degrad 8:1–19. 10.1038/s41529-024-00540-z 10.1038/s41529-024-00540-z PMC11618075 39649129

[CR38] Harris RL, Schuerger AC (2025) Hydrogenotrophic methanogenesis at 7–12 mbar by *Methanosarcina barkeri* under simulated Martian atmospheric conditions. Sci Rep 15:2880. 10.1038/s41598-025-86145-1 39843490 10.1038/s41598-025-86145-1 PMC11754898

[CR39] Hausmannová L, Dohnálková M, Matušková E, Lahodová Z, Augusta J (2023) Technical design of the deep geological repository 2023 (No. Technical report 711/2023/ENG). SÚRAO

[CR40] Havlová V, Prchal K, Hofmanová E, Dobrev D, Večerník P, Svoboda K, Trpkošová D, Trtílek R, Vojtěchová H, Bukovská Z, Franěk J, Mixa P, Pertoldová J, Švagera O, Hroch T, Nývlt D, Rataj J, Vetešník A, Svoboda J, Milický M, Uhlík J, Hokr M, Maryška J, Říha J, Steinová J, Staš L, Hasal M, Blaheta R (2020) Research support for the safety assessment of the deep geological repository final report. SÚRAO, Prague. (Final report No. 462/2020/EN)

[CR41] Haynes HM, Bailey MT, Lloyd JR (2021) Bentonite barrier materials and the control of microbial processes: safety case implications for the geological disposal of radioactive waste. Chem Geol 581:120353. 10.1016/j.chemgeo.2021.120353

[CR42] Holmes DE, Rotaru A-E, Ueki T, Shrestha PM, Ferry JG, Lovley DR (2018) Electron and proton flux for carbon dioxide reduction in *Methanosarcina barkeri* during direct interspecies electron transfer. Front Microbiol. 10.3389/fmicb.2018.03109 30631315 10.3389/fmicb.2018.03109 PMC6315138

[CR43] Huang Y, Xu D, Huang L, Lou Y, Muhadesi J-B, Qian H, Zhou E, Wang B, Li X-T, Jiang Z, Liu S-J, Zhang D, Jiang C-Y (2021) Responses of soil microbiome to steel corrosion. Npj Biofilms Microbiomes 7:1–13. 10.1038/s41522-020-00175-3 33479252 10.1038/s41522-020-00175-3 PMC7820017

[CR44] IAEA (2009) Classification of Radioactive Waste: General Safety Guide. IAEA, Austria, Vienna

[CR45] IAEA (2021) Nuclear energy for a net zero world. IAEA, Austria, Vienna

[CR46] Jang J, Lee M, Kim G-Y, Yoon S (2025) Long-term performance test of Cu canisters used for disposing of spent nuclear fuels under aerobic groundwater. Nucl Eng Technol 57:103678. 10.1016/j.net.2025.103678

[CR47] Kašpar V, Šachlová Š, Hofmanová E, Komárková B, Havlová V, Aparicio C, Černá K, Bartak D, Hlaváčková V (2021) Geochemical, geotechnical, and microbiological changes in Mg/Ca bentonite after thermal loading at 150°C. Minerals 11:965. 10.3390/min11090965

[CR48] Kato S, Yumoto I, Kamagata Y (2015) Isolation of acetogenic bacteria that induce biocorrosion by utilizing metallic iron as the sole electron donor. Appl Environ Microbiol 81:67–73. 10.1128/AEM.02767-14 25304512 10.1128/AEM.02767-14 PMC4272740

[CR49] Kaufhold S, Dohrmann R, Wallis I, Weber C (2023) Chemical and mineralogical reactions of bentonites in geotechnical barriers at elevated temperatures: review of experimental evidence and modelling progress. Clay Min 58:280–300. 10.1180/clm.2023.26

[CR50] Kim J, Dong H, Seabaugh J, Newell SW, Eberl DD (2004) Role of microbes in the smectite-to-illite reaction. Science 303:830–832. 10.1126/science.1093245 14764877 10.1126/science.1093245

[CR51] King F (2013) Container materials for the storage and disposal of nuclear waste. Corrosion 69:986–1011. 10.5006/0894

[CR52] King F, Kolàř M, Briggs S, Behazin M, Keech P, Diomidis N (2024) Review of the modelling of corrosion processes and lifetime prediction for HLW/SF containers—Part 2: performance assessment models. Corros Mater Degrad 5:289–339. 10.3390/cmd5020013

[CR53] Knisz J, Eckert R, Gieg LM, Koerdt A, Lee JS, Silva ER, Skovhus TL, Stepec A, Wade BA, A S (2023) Microbiologically influenced corrosion—more than just microorganisms. FEMS Microbiol Rev 47:fuad041. 10.1093/femsre/fuad041 37437902 10.1093/femsre/fuad041 PMC10479746

[CR54] Koblitz J, Halama P, Spring S, Thiel V, Baschien C, Hahnke RL, Pester M, Overmann J, Reimer LC (2023) MediaDive: the expert-curated cultivation media database. Nucleic Acids Res 51:D1531–D1538. 10.1093/nar/gkac803 36134710 10.1093/nar/gkac803 PMC9825534

[CR55] Kotsyurbenko OR, Glagolev MV, Nozhevnikova AN, Conrad R (2001) Competition between homoacetogenic bacteria and methanogenic archaea for hydrogen at low temperature. FEMS Microbiol Ecol 38:153–159. 10.1111/j.1574-6941.2001.tb00893.x

[CR56] Kumpulainen S, Hagros A, Ville H (2022) State-of-the-art study of foreign concepts of engineered elements in DGR (Technical Report No. 575/2022). SÚRAO, Praha, Czech Republic

[CR57] Lang LZ, Baille W, Tripathy S, Schanz T (2018) Experimental study on the influence of preliminary desiccation on the swelling pressure and hydraulic conductivity of compacted bentonite. Clay Min 53:733–744. 10.1180/clm.2018.53

[CR58] Lhotský O, Kukačka J, Slunský J, Marková K, Němeček J, Knytl V, Cajthaml T (2021) The effects of hydraulic/pneumatic fracturing-enhanced remediation (FRAC-IN) at a site contaminated by chlorinated ethenes: a case study. J Hazard Mater 417:125883. 10.1016/j.jhazmat.2021.125883 33971551 10.1016/j.jhazmat.2021.125883

[CR60] Libert M, Bildstein O, Esnault L, Jullien M, Sellier R (2011) Molecular hydrogen: An abundant energy source for bacterial activity in nuclear waste repositories. Phys Chem Earth Parts ABC 36:1616–1623. Clays in Natural & Engineered Barriers for Radioactive Waste Confinement10.1016/j.pce.2011.10.010

[CR61] Lilja C (2010) Corrosion calculations report for the safety assessment SR-Site (No. Technical Report TR-10-66). Svensk kärnbränslehantering (SKB)

[CR59] Li Q, Gong L, Chen X-G, Gadd GM, Liu D (2025) Dual role of microorganisms in metal corrosion: a review of mechanisms of corrosion promotion and inhibition. Front Microbiol 16. 10.3389/fmicb.2025.1552103 10.3389/fmicb.2025.1552103 PMC12017684 40270819

[CR63] Liu B, Sun M, Lu F, Du C, Li X (2021) Study of biofilm-influenced corrosion on X80 pipeline steel by a nitrate-reducing bacterium, *Bacillus cereus*, in artificial Beijing soil. Colloids Surf B Biointerfaces 197:111356. 10.1016/j.colsurfb.2020.111356 33007505 10.1016/j.colsurfb.2020.111356

[CR62] Liu H, Meng G, Li W, Gu T, Liu, H (2019) Microbiologically influenced corrosion of carbon steel beneath a deposit in CO2-saturated formation water containing desulfotomaculum nigrificans. Front Microbiol 10. 10.3389/fmicb.2019.01298 10.3389/fmicb.2019.01298 PMC6581712 31244809

[CR65] Liu P, Zhang H, Fan Y, Xu D (2023) Microbially influenced corrosion of steel in marine environments: a review from mechanisms to prevention. Microorganisms 11:2299. 10.3390/microorganisms11092299 37764143 10.3390/microorganisms11092299 PMC10535020

[CR64] Liu W, Zhou Z, Li Z, Li M, Li Q, Ye Z, Yao J, Zhong X (2022) The high-temperature mechanical properties of HS110S steel and its corrosion behaviors in harsh downhole environment. Anti-Corros Methods Mater 69:550–560. 10.1108/ACMM-05-2022-2650

[CR66] Logan BE (2009) Exoelectrogenic bacteria that power microbial fuel cells. Nat Rev Microbiol 7:375–381. 10.1038/nrmicro2113 19330018 10.1038/nrmicro2113

[CR67] Ma J, Pekala M, Alt-Epping P, Pastina B, Maanoja S, Wersin P (2022) 3D modelling of long-term sulfide corrosion of copper canisters in a spent nuclear fuel repository. Appl Geochem 146:105439. 10.1016/j.apgeochem.2022.105439

[CR68] Marschall P, Talandier J, Kolditz O (2024) Barrier integrity: gas-induced impacts and model-based interpretation. Task 3. Final technical report (No. Deliverable D6.8). GAS WP Eurad

[CR69] Marshall MHM, McKelvie JR, Simpson AJ, Simpson MJ (2015) Characterization of natural organic matter in bentonite clays for potential use in deep geological repositories for used nuclear fuel. Appl Geochem 54:43–53. 10.1016/j.apgeochem.2014.12.013

[CR70] Martinez Abizu P (2020) pairwiseAdonis: Pairwise multilevel comparison using adonis. R package version 0.4

[CR71] Martinez-Moreno MF, Povedano-Priego C, Mumford AD, Morales-Hidalgo M, Mijnendonckx K, Jroundi F, Ojeda JJ, Merroun ML (2024) Microbial responses to elevated temperature: evaluating bentonite mineralogy and copper canister corrosion within the long-term stability of deep geological repositories of nuclear waste. Sci Total Environ 915:170149. 10.1016/j.scitotenv.2024.170149 38242445 10.1016/j.scitotenv.2024.170149

[CR72] Masurat P, Eriksson S, Pedersen K (2010) Evidence of indigenous sulphate-reducing bacteria in commercial Wyoming bentonite MX-80. Appl Clay Sci 47:51–57. 10.1016/j.clay.2008.07.002

[CR73] McMurdie PJ, Holmes S (2013) Phyloseq: an R package for reproducible interactive analysis and graphics of microbiome census data. PLoS One 8:e61217. 10.1371/journal.pone.0061217 23630581 10.1371/journal.pone.0061217 PMC3632530

[CR74] Medina D, Walke JB, Gajewski Z, Becker MH, Swartwout MC, Belden LK (2017) Culture media and individual hosts affect the recovery of culturable bacterial diversity from amphibian skin. Front Microbiol 8:1574. 10.3389/fmicb.2017.01574 28883811 10.3389/fmicb.2017.01574 PMC5573730

[CR75] Messina E, Pascucci M, Riccucci C, Boccaccini F, Blanco-Valera MT, Garcia-Lodeiro I, Ingo GM, Di Carlo G (2023) Smart inhibition action of amino acid-modified layered double hydroxide and its application on carbon steel. Molecules 28:5863. 10.3390/molecules28155863 37570833 10.3390/molecules28155863 PMC10421257

[CR76] Necib S, Diomidis N, Keech P, Nakayama M (2017) Corrosion of carbon steel in clay environments relevant to radioactive waste geological disposals, Mont Terri rock laboratory (Switzerland). Swiss J Geosci 110:329–342. 10.1007/s00015-016-0259-7

[CR77] Ning J, Zheng Y, Young D, Brown B, Nešić S (2013) Thermodynamic study of hydrogen sulfide corrosion of mild steel. Corrosion 70:375–389. 10.5006/0951

[CR78] Noh D-H, Kim S, Eun J, Kim Y-R (2025) Experimental study on gas permeability and desiccation cracking of fiber-reinforced compacted bentonite at high temperature. Constr Build Mater 478:141053. 10.1016/j.conbuildmat.2025.141053

[CR79] Ohazuruike L, Lee KJ (2023) A comprehensive review on clay swelling and illitization of smectite in natural subsurface formations and engineered barrier systems. Nucl Eng Technol 55:1495–1506. 10.1016/j.net.2023.01.007

[CR80] Palaniappan B, Toleti SR (2016) Characterization of microfouling and corrosive bacterial community of a firewater distribution system. J Biosci Bioeng 121:435–441. 10.1016/j.jbiosc.2015.08.009 26467696 10.1016/j.jbiosc.2015.08.009

[CR81] Pont A, Coene E, Idiart A (2020) Bentonite erosion project – Preliminary study for the numerical simulation of bentonite erosion (No. SKB P-20-16). Svensk kärnbränslehantering (SKB).

[CR82] Posiva, Oy (2017) Safety evaluation for a KBS-3H spent nuclear fuel repository at Olkiluoto – Features, Events and Processes. POSIVA 2016-03. POSIVA.

[CR83] Pospiskova I, Dobrev D, Kouril M, Stoulil J, Novikova D, Kotnour P, Matal O (2017) Czech national programme and disposal canister concept. Corros Eng Sci Technol 52:6–10. 10.1080/1478422X.2017.1300379

[CR84] Quast C, Pruesse E, Yilmaz P, Gerken J, Schweer T, Yarza P, Peplies J, Glöckner FO (2013) The SILVA ribosomal RNA gene database project: improved data processing and web-based tools. Nucleic Acids Res 41:D590–D596. 10.1093/nar/gks1219 23193283 10.1093/nar/gks1219 PMC3531112

[CR85] Rajala P, Carpén L, Vepsäläinen M, Raulio M, Sohlberg E, Bomberg M (2015) Microbially induced corrosion of carbon steel in deep groundwater environment. Front Microbiol. 10.3389/fmicb.2015.00647 26257707 10.3389/fmicb.2015.00647 PMC4513438

[CR86] Rajala P, Huttunen-Saarivirta E, Bomberg M, Carpén L (2019) Corrosion and biofouling tendency of carbon steel in anoxic groundwater containing sulphate reducing bacteria and methanogenic archaea. Corros Sci 159:108148

[CR87] Ruan X, Yang L, Wang Y, Dong Y, Xu D, Zhang M (2023) Biofilm-induced corrosion inhibition of Q235 carbon steel by *Tenacibaculum mesophilum* D-6 and *Bacillus* sp. Y-6. Metals 13:649. 10.3390/met13040649

[CR88] Salgar-Chaparro SJ, Lepkova K, Pojtanabuntoeng T, Darwin A, Machuca LL (2020) Nutrient level determines biofilm characteristics and subsequent impact on microbial corrosion and biocide effectiveness. Appl Environ Microbiol 86:e02885–e02819. 10.1128/AEM.02885-19 31980429 10.1128/AEM.02885-19 PMC7082584

[CR89] Sattar S, Hussain A, Qazi JI, Javid A, Mehmood S (2024) Sulfate reducing bacterial corrosion of mild steel in liquid and solid media. Pak J Zool 56:1351–1357. 10.17582/journal.pjz/20220713160735

[CR90] Scardino A, Mangiaterra G, Citterio B, Hijazi S, Ciacci C, Fehervari M, Frangipani E (2025) Cinnamaldehyde effectively disrupts Desulfovibrio vulgaris biofilms: potential implication to mitigate microbiologically influenced corrosion. Appl Environ Microbiol 91:e02200–e02224. 10.1128/aem.02200-24 40293245 10.1128/aem.02200-24 PMC12093973

[CR91] Schmidt O, Hink L, Horn MA, Drake HL (2016) Peat: home to novel syntrophic species that feed acetate- and hydrogen-scavenging methanogens. ISME J 10:1954–1966. 10.1038/ismej.2015.256 26771931 10.1038/ismej.2015.256 PMC5029166

[CR92] Schwab L, Rago L, Koch C, Harnisch F (2019) Identification of *Clostridium cochlearium* as an electroactive microorganism from the mouse gut microbiome. Bioelectrochemistry 130:107334. 10.1016/j.bioelechem.2019.107334 31352302 10.1016/j.bioelechem.2019.107334

[CR93] Sellin P (2017) BELBaR: Bentonite erosion: effects on the long term performance of the engineered barrier and radionuclide transport (Final report). Svensk kärnbränslehantering (SKB)

[CR95] Shrestha R, Cerna K, Spanek R, Bartak D, Cernousek T, Sevcu A (2022) The effect of low-pH concrete on microbial community development in bentonite suspensions as a model for microbial activity prediction in future nuclear waste repository. Sci Total Environ 808:151861. 10.1016/j.scitotenv.2021.151861 34838551 10.1016/j.scitotenv.2021.151861

[CR94] Shrestha R, Černoušek T, Stoulil J, Kovářová H, Sihelská K, Špánek R, Ševců A, Steinová J (2021) Anaerobic microbial corrosion of carbon steel under conditions relevant for deep geological repository of nuclear waste. Sci Total Environ 800:149539. 10.1016/j.scitotenv.2021.149539 34392220 10.1016/j.scitotenv.2021.149539

[CR97] SKB (2010a) Design and production of the KBS-3 repository (Technical Report No. TR-10-12). Svensk kärnbränslehantering (SKB)

[CR96] SKB, (2010b) Buffer, backfill and closure process report for the safety assessment SR-Site (No. TR-10-47). Svensk kärnbränslehantering (SKB).

[CR98] Svensson D, Dueck A, Nilsson U, Olsson S, Sandén T, Lydmark S, Jägerwall S, Pedersen K, Hansen S (2011) Alternative buffer material. Status of the ongoing laboratory investigation of reference materials and test package 1. (No. TR-11-06). Swedish Nuclear Fuel and Waste Management Co, Stockholm, Sweden

[CR99] Svoboda J, Šťástka J, Vašíček R, Špinka O, Bureš P, Pospíšková I, Vozár M, Krajňák M, Šachlová Š, Večerník P, Zuna M, Havlová V, Stiblíková P, Černá K Hlaváčková, Veronika, 2022. Design of the Czech concept for the buffer, backfill, plugs, and sealing of disposal chambers for other RAW; the sealing of other underground spaces and construction elements (No. Technical report 644/2022/ENG). SÚRAO

[CR100] Taghavi Kalajahi S, Misra A, Koerdt A (2024) Nanotechnology to mitigate microbiologically influenced corrosion (MIC). Front Nanotechnol. 10.3389/fnano.2024.1340352

[CR101] Telford WM, Geldart LP, Sheriff RE (1990) Applied Geophysics. Cambridge University Press

[CR102] Turnbull A (2009) A review of the possible effects of hydrogen on lifetime of carbon steel nuclear waste canisters (No. Technical Report NTB 09 – 04:). NAGRA

[CR103] Ueki T, Nevin KP, Woodard TL, Aklujkar MA, Holmes DE, Lovley DR (2018) Construction of a *Geobacter* strain with exceptional growth on cathodes. Front Microbiol. 10.3389/fmicb.2018.01512 30057572 10.3389/fmicb.2018.01512 PMC6053493

[CR104] Vachon MA, Engel K, Beaver RC, Slater GF, Binns WJ, Neufeld JD (2021) Fifteen shades of clay: distinct microbial community profiles obtained from bentonite samples by cultivation and direct nucleic acid extraction. Sci Rep 11:22349. 10.1038/s41598-021-01072-1 34785699 10.1038/s41598-021-01072-1 PMC8595889

[CR106] Wang X-W, Sun Z, Jia H, Michel-Mata S, Angulo MT, Dai L, He X, Weiss ST, Liu Y-Y (2024) Identifying keystone species in microbial communities using deep learning. Nat Ecol Evol 8:22–31. 10.1038/s41559-023-02250-2 37974003 10.1038/s41559-023-02250-2 PMC12125608

[CR105] Wan H, Song D, Zhang D, Du C, Xu D, Liu Z, Ding D, Li X (2018) Corrosion effect of *Bacillus cereus* on X80 pipeline steel in a Beijing soil environment. Bioelectrochemistry 121:18–26. 10.1016/j.bioelechem.2017.12.011 29329018 10.1016/j.bioelechem.2017.12.011

[CR107] Xu L, Khan A, Wang S, Kijkla P, Kumseranee S, Punpruk S, Gu T (2024) Preliminary investigations of microbiologically influenced corrosion of 304 stainless steel by anaerobic *Clostridioides difficile* biofilm. Int Biodeterior Biodegrad 194:105871. 10.1016/j.ibiod.2024.105871

[CR108] Zani ACB, de Souza JC, de Andrade AR, Reginatto V (2024) Neutral Red film augments extracellular electron transfer performed by *Clostridium pasteurianum* DSM 525. Fermentation 10:497. 10.3390/fermentation10100497

