# Supplementary material for: Microbiologically influenced corrosion (MIC) potential of bentonite microorganisms: implications for a deep geological repository for nuclear waste
Source: World J Microbiol Biotechnol. 2026 Jun 1;42(6):321. doi: 10.1007/s11274-026-05039-0 (PMC13226343; doi:10.1007/s11274-026-05039-0)
Supplement: Supplementary file 1 — Supplementary Material 1 (DOCX 63.8 KB) [file 11274_2026_5039_MOESM1_ESM.docx]

**Microbiologically influenced corrosion (MIC) potential of bentonite microorganisms: implications for a deep geological repository for nuclear waste**

**Supplementary Material 2**

Kateřina Černá^1*^, Saqlain Saqib Mukhtar^2^, Richard Bureš^2^, Gabriela Alfaro-Espinoza^3^, Andrea Koerdt^3^, Jakub Říha^1^, Veronika Hlavackova^1^, Jan Stoulil^2^

1 Technical University of Liberec, Institute for Nanomaterials, Advanced Technologies and Innovation, Studenstká 1402/2, 46117 Liberec, Czech Republic

2 University of Chemistry and Technology, Technická 5, 166 28 Prague, Czech Republic

3 Bundesanstalt für Materialforschung und -prüfung (BAM), Unter den Eichen 87, 12205 Berlin, Germany

*Corresponding author: [katerina.cerna1@tul.cz](mailto:katerina.cerna1@tul.cz), ORCID ID: 0000-0003-3351-6372

**Supplementary Table S1: Chemical composition of Trace element solution SL10 and Wolins vitamin solution used in medium ME120.**

| **Compound** | **Amount** | **Unit** |
| --- | --- | --- |
| **Trace element solution SL-10** |  |  |
| HCl (25%) | 10 | mL |
| FeCl2 x 4 H2O | 1,5 | g |
| ZnCl2 | 70 | mg |
| MnCl2 x 4 H2O | 100 | mg |
| H3BO3 | 6 | mg |
| CoCl2 x 6 H2O | 190 | mg |
| CuCl2 x 2 H2O | 2 | mg |
| NiCl2 x 6 H2O | 24 | mg |
| Na2MoO4 x 2 H2O | 36 | mg |
| Distilled water | 990 | mL |
|  |  |  |
| **Wolins vitamins solution** |  |  |
| [Biotin](https://bacmedia.dsmz.de/ingredients/46) | 2 | mg |
| [Folic acid](https://bacmedia.dsmz.de/ingredients/138) | 2 | mg |
| [Pyridoxine hydrochloride](https://bacmedia.dsmz.de/ingredients/519) | 10 | mg |
| [Thiamine HCl](https://bacmedia.dsmz.de/ingredients/1207) | 5 | mg |
| [Riboflavin](https://bacmedia.dsmz.de/ingredients/136) | 5 | mg |
| [Nicotinic acid](https://bacmedia.dsmz.de/ingredients/139) | 5 | mg |
| [Calcium D-(+)-pantothenate](https://bacmedia.dsmz.de/ingredients/1512) | 5 | mg |
| [Vitamin B12](https://bacmedia.dsmz.de/ingredients/18) | 0,1 | mg |
| [p-Aminobenzoic acid](https://bacmedia.dsmz.de/ingredients/47) | 5 | mg |
| [(DL)-alpha-Lipoic acid](https://bacmedia.dsmz.de/ingredients/252) | 5 | mg |
| [Distilled water](https://bacmedia.dsmz.de/ingredients/4) | 1000 | mL |

**Supplementary Table S2: Taxa identified as contaminants.** Statistical identification and removal of contaminant sequences using the Decontam package v1.20.0 (Davis et al., 2018).

| Kingdom | Phylum | Order | Family | Genus |
| --- | --- | --- | --- | --- |
| d__Bacteria | Pseudomonadota | Enterobacterales | Pasteurellaceae | Haemophilus |
| d__Bacteria | Pseudomonadota | Burkholderiales | Neisseriaceae | NA |
| d__Bacteria | Bacillota | Lactobacillales | Lactobacillaceae | Lactobacillus |
| d__Bacteria | Pseudomonadota | Pseudomonadales | Pseudomonadaceae | Thiopseudomonas |
| d__Bacteria | Thermodesulfobacteriota | Geobacterales | Geobacteraceae | Pelotalea |
| d__Bacteria | Actinomycetota | Frankiales | Sporichthyaceae | hgcI_clade |
| d__Bacteria | Bacteroidota | Flavobacteriales | Weeksellaceae | Chryseobacterium |
| d__Bacteria | Bacillota | Lactobacillales | Enterococcaceae | Enterococcus |
| d__Bacteria | Bacillota | Staphylococcales | Staphylococcaceae | Staphylococcus |
| d__Bacteria | Pseudomonadota | Pseudomonadales | Pseudomonadaceae | Pseudomonas |
| d__Bacteria | Pseudomonadota | Enterobacterales | Enterobacteriaceae | Escherichia-Shigella |
| d__Bacteria | Bacteroidota | Bacteroidales | Prevotellaceae | NA |
| d__Bacteria | Pseudomonadota | Burkholderiales | Hydrogenophilaceae | Hydrogenophilus |
| d__Bacteria | Bacillota | Veillonellales-Selenomonadales | Veillonellaceae | Veillonella |
| d__Bacteria | Deinococcota | Thermales | Thermaceae | Thermus |
| d__Bacteria | Pseudomonadota | Hyphomicrobiales | Xanthobacteraceae | Bradyrhizobium |
| d__Bacteria | Pseudomonadota | Enterobacterales | Enterobacteriaceae | NA |
| d__Bacteria | Pseudomonadota | Lysobacterales | Lysobacteraceae | Stenotrophomonas |
| d__Bacteria | Pseudomonadota | Enterobacterales | Alteromonadaceae | Alishewanella |
| d__Bacteria | Pseudomonadota | Burkholderiales | Alcaligenaceae | Paracandidimonas |
| d__Bacteria | Pseudomonadota | Sphingomonadales | Sphingomonadaceae | Sphingomonas |
| d__Bacteria | Actinomycetota | Micrococcales | Micrococcaceae | Paenarthrobacter |
| d__Bacteria | Pseudomonadota | Lysobacterales | Lysobacteraceae | Vulcaniibacterium |
| d__Bacteria | Pseudomonadota | Pseudomonadales | NA | NA |
| d__Bacteria | Pseudomonadota | Rhodobacterales | Paracoccaceae | NA |
| d__Bacteria | Actinomycetota | Micrococcales | Micrococcaceae | Micrococcus |
| d__Bacteria | Actinomycetota | Propionibacteriales | Propionibacteriaceae | Cutibacterium |
| d__Bacteria | Bacillota | Lactobacillales | Carnobacteriaceae | Granulicatella |
| d__Bacteria | Bacteroidota | Flavobacteriales | Weeksellaceae | Moheibacter |
| d__Bacteria | Pseudomonadota | Lysobacterales | Lysobacteraceae | Xanthomonas |
| d__Bacteria | Actinomycetota | Micrococcales | Micrococcaceae | Rothia |
| d__Bacteria | Bacillota | Peptostreptococcales-Tissierellales | Family_XI | Anaerococcus |
| d__Bacteria | Actinomycetota | Micrococcales | Micrococcaceae | Kocuria |
| d__Bacteria | Actinomycetota | Mycobacteriales | Nocardiaceae | Rhodococcus |
| d__Bacteria | Pseudomonadota | Burkholderiales | Comamonadaceae | NA |
| d__Bacteria | Pseudomonadota | Hyphomicrobiales | Rhizobiaceae | NA |
| d__Bacteria | Pseudomonadota | Burkholderiales | Comamonadaceae | Comamonas |
| d__Bacteria | Pseudomonadota | Enterobacterales | Vibrionaceae | Photobacterium |
| d__Bacteria | Pseudomonadota | Lysobacterales | Lysobacteraceae | Pseudoxanthomonas |
| d__Bacteria | Pseudomonadota | Enterobacterales | Psychromonadaceae | Psychromonas |
| d__Bacteria | Bacteroidota | Sphingobacteriales | Sphingobacteriaceae | Pedobacter |
| d__Bacteria | Pseudomonadota | Caulobacterales | Caulobacteraceae | Brevundimonas |
| d__Bacteria | Pseudomonadota | Burkholderiales | Comamonadaceae | Caldimonas |
| d__Bacteria | Actinomycetota | Mycobacteriales | Corynebacteriaceae | Corynebacterium |
| d__Bacteria | Pseudomonadota | Pseudomonadales | Moraxellaceae | Enhydrobacter |
| d__Bacteria | Bacillota | Lactobacillales | Streptococcaceae | Lactococcus |
| d__Bacteria | Pseudomonadota | Rhodobacterales | Paracoccaceae | Paracoccus |
| d__Bacteria | Pseudomonadota | Burkholderiales | Burkholderiaceae | Cupriavidus |
| d__Bacteria | Pseudomonadota | Burkholderiales | Neisseriaceae | Neisseria |
| d__Bacteria | Actinomycetota | Mycobacteriales | Corynebacteriaceae | Lawsonella |
| d__Bacteria | Actinomycetota | Micrococcales | Dermacoccaceae | Dermacoccus |
| d__Bacteria | Pseudomonadota | Enterobacterales | Pseudoalteromonadaceae | Pseudoalteromonas |
| d__Bacteria | Pseudomonadota | Pseudomonadales | Cellvibrionaceae | Cellvibrio |
| d__Bacteria | Bacillota | Peptostreptococcales-Tissierellales | Family_XI | Finegoldia |
| d__Bacteria | Pseudomonadota | Enterobacterales | Vibrionaceae | Vibrio |
| d__Bacteria | Bacteroidota | Cytophagales | Spirosomataceae | Spirosoma |
| d__Bacteria | Bacillota | Lactobacillales | Lactobacillaceae | Latilactobacillus |
| d__Bacteria | Pseudomonadota | Enterobacterales | Yersiniaceae | NA |
| d__Bacteria | Bacteroidota | Flavobacteriales | Weeksellaceae | Cloacibacterium |
| d__Bacteria | Actinomycetota | Micrococcales | Intrasporangiaceae | NA |
| d__Bacteria | Bacillota | Peptostreptococcales-Tissierellales | Family_XI | Peptoniphilus |
| d__Bacteria | Pseudomonadota | Hyphomicrobiales | Beijerinckiaceae | Lichenibacterium |
| d__Bacteria | Pseudomonadota | Burkholderiales | Comamonadaceae | Tepidimonas |
| d__Bacteria | Pseudomonadota | Hyphomicrobiales | Beijerinckiaceae | Methylobacterium |
| d__Bacteria | Bacillota | Lactobacillales | Streptococcaceae | Streptococcus |
| d__Bacteria | Pseudomonadota | Burkholderiales | Comamonadaceae | Rhodoferax |
| d__Bacteria | Bacteroidota | Flavobacteriales | Flavobacteriaceae | Flavobacterium |
| d__Bacteria | Patescibacteria | Candidatus_Zambryskibacteria | NA | NA |
| d__Bacteria | Pseudomonadota | Hyphomicrobiales | Beijerinckiaceae | Methylorubrum |
| d__Bacteria | Pseudomonadota | Pseudomonadales | Moraxellaceae | Acinetobacter |
| d__Bacteria | Actinomycetota | Actinomycetales | Actinomycetaceae | Schaalia |
| d__Bacteria | Actinomycetota | Propionibacteriales | Nocardioidaceae | NA |
| d__Bacteria | Chloroflexota | NA | NA | NA |
| d__Bacteria | Actinomycetota | Actinomycetales | Actinomycetaceae | Flaviflexus |
| d__Bacteria | Pseudomonadota | Lysobacterales | Rhodanobacteraceae | Luteibacter |
| d__Bacteria | Actinomycetota | Micrococcales | Brevibacteriaceae | Brevibacterium |

**Supplementary Table S3: Differences in corrosion rates (g.m^-2^.a^-1^) between abiotic and inoculated samples in Batch Experiment 1.** Welch two-sample T-test comparison between different samples and their respective sterile controls in Batch Experiment 1. LB - Lysogeny broth, ME - medium 120 (Koblitz et al., 2023), NRM - Nitrate Broth, PGM - Postgate medium, PW - synthetic bentonite pore water, PWN - PW amended with nitrate, R2A - R2A medium. Sterile - mean corrosion rates in sterile control samples; 100/25/0 - respective media with 100 %/25 %/0 % of organic content compared to the original prescription. P - samples with powered iron.

| **LB** | **Mean biotic** | **Mean sterile** | **t-value** | **df** | **p-value** | **Significance** |
| --- | --- | --- | --- | --- | --- | --- |
| LB100.P vs sterile | 169 | 132.6 | 1.025 | 8.00 | 0.335 | No |
| LB100 vs sterile | 191.5 | 132.6 | 0.916 | 4.46 | 0.406 | No |
| LB25.P vs sterile | 257.3 | 132.6 | 2.386 | 5.43 | 0.059 | No(boredline) |
| LB25 vs sterile | 163.1 | 132.6 | 0.594 | 5.55 | 0.576 | No |
|  |  |  |  |  |  |  |
| **ME** | **Mean biotic** | **Mean sterile** | **t-value** | **df** | **p-value** | **Significance** |
| ME100.P vs sterile | 90.4 | 368.7 | -6.443 | 5.17 | 0.001 | Yes |
| ME100 vs sterile | 268.1 | 368.7 | -2.337 | 5.11 | 0.066 | No (borderline) |
| ME25.P vs sterile | 140.9 | 368.7 | -3.640 | 7.24 | 0.008 | Yes |
| ME25 vs sterile | 123.3 | 368.7 | -5.645 | 5.31 | 0.002 | Yes |
|  |  |  |  |  |  |  |
| **NRM** | **Mean biotic** | **Mean sterile** | **t-value** | **df** | **p-value** | **Significance** |
| NRM100.P vs sterile | 165.3 | 191.5 | -0.620 | 5.69 | 0.559 | No |
| MRM100 vs sterile | 120.4 | 191.5 | -2.688 | 6.99 | 0.031 | Yes |
| NRM25.P vs sterile | 106.9 | 191.5 | -3.102 | 7.42 | 0.016 | Yes |
| NRM25 vs sterile | 258.0 | 191.5 | 0.812 | 3.56 | 0.468 | No |
| NRM0.P vs sterile | 259.5 | 191.5 | 1.751 | 6.31 | 0.128 | No |
| NRM0 vs sterile | 252.1 | 191.5 | 1.029 | 4.19 | 0.359 | No |
|  |  |  |  |  |  |  |
| **PGM** | **Mean biotic** | **Mean sterile** | **t-value** | **df** | **p-value** | **Significance** |
| PGM100.P vs sterile | 57.8 | 103.4 | -1.577 | 7.91 | 0.154 | No |
| PGM100 vs sterile | 104.9 | 103.4 | 0.060 | 6.23 | 0.954 | No |
| PGM25.P vs sterile | 54.8 | 103.4 | -1.992 | 5.51 | 0.098 | No |
| PGM25 vs sterile | 79.1 | 103.4 | -1.012 | 5.19 | 0.356 | No |
| PGM0.P vs sterile | 144.5 | 103.4 | 1.597 | 6.49 | 0.158 | No |
| PGM0 vs sterile | 203.8 | 103.4 | 3.066 | 7.7 | 0.016 | Yes |
|  |  |  |  |  |  |  |
| **R2A** | **Mean biotic** | **Mean sterile** | **t-value** | **df** | **p-value** | **Significance** |
| R2A100.P vs sterile | 91.9 | 85.6 | 0.183 | 7.25 | 0.860 | No |
| R2A100 vs sterile | 110.2 | 85.6 | 0.665 | 6.68 | 0.528 | No |
| R2A25.P vs sterile | 86.7 | 85.6 | 0.028 | 6.37 | 0.978 | No |
| R2A25 vs sterile | 176.0 | 85.6 | 3.718 | 5.58 | 0.011 | Yes |
| R2A0.P vs sterile | 191.6 | 85.6 | 1.622 | 3.92 | 0.182 | No |
| R2A0 vs sterile | 123.8 | 85.6 | 0.819 | 5.06 | 0.450 | No |
|  |  |  |  |  |  |  |
| **PW** | **Mean biotic** | **Mean sterile** | **t-value** | **df** | **p-value** | **Significance** |
| PW.P vs sterile | 220.3 | 84.1 | 2.139 | 3.55 | 0.108 | No |
| PW vs sterile | 154 | 84.1 | 1.335 | 3.86 | 0.255 | No |
|  |  |  |  |  |  |  |
| **PWN** | **Mean biotic** | **Mean sterile** | **t-value** | **df** | **p-value** | **Significance** |
| PWN.P vs sterile | 140.4 | 153 | –0.153 | 5.10 | 0.884 | No |
| PWN vs sterile | 307.3 | 153 | 1.856 | 5.03 | 0.122 | No |

**Supplementary Table S4: Differences in corrosion rates between Batch Experiments 1 and 2 across all media (all) and in respective media types.** Wilcoxon signed-rank test or Paired t-test was applied based on the data normality prior check. LB - Lysogeny broth, ME - medium 120 (Koblitz et al., 2023), NRM - Nitrate Broth, PGM - Postgate medium, PW - synthetic bentonite pore water, PWN - PW amended with nitrate, R2A - R2A medium.

| **Medium** | **Test Type** | **Test Statistic** | **df** | **p-value** | **Significant (p < 0.05)** |
| --- | --- | --- | --- | --- | --- |
| All | Wilcoxon signed-rank | V = 824 | – | 0.5053 | No |
| LB | Wilcoxon signed-rank | V = 29 | – | 0.1484 | No |
| ME | Wilcoxon signed-rank | V = 17 | – | 0.9453 | No |
| NRM | Wilcoxon signed-rank | V = 27 | – | 0.3804 | No |
| PGM | Paired t-test | t = –2.1263 | 11 | 0.0569 | No (trend, p ≈ 0.057) |
| PW/PWN | Wilcoxon signed-rank | V = 30 | – | 0.1094 | No |
| R2A | Paired t-test | t = –2.2188 | 11 | 0.0485 | Yes |

**Supplementary Table S5: pairwise comparison of Bulk medium vs. Swab samples diversity across different media types.** Pairwise PERMANOVA was applied*.* LB - Lysogeny broth, ME - medium 120 (Koblitz et al., 2023), NRM - Nitrate Broth, PGM - Postgate medium, PW - synthetic bentonite pore water, R2A - R2A medium. No genetic analyses of swabs were performed in PW/PWN medium samples because of the low biomass that was obtained.

| **Medium** | **Pair** | **Df** | **Sum of Squares** | **F-value** | **R²** | **p-adjusted** | **Significant**  **(p < 0.05)** |
| --- | --- | --- | --- | --- | --- | --- | --- |
| LB | medium vs swab | 1 | 0.198 | 1.322 | 0.0484 | 0.236 | No |
| ME | medium vs swab | 1 | 0.361 | 1.748 | 0.0551 | 0.112 | No |
| NRM | medium vs swab | 1 | 0.157 | 0.698 | 0.0227 | 0.705 | No |
| PGM | medium vs swab | 1 | 1.061 | 3.607 | 0.1073 | 0.001 | Yes |
| R2A | medium vs swab | 1 | 0.672 | 3.981 | 0.1328 | 0.003 | Yes |

**Supplementary Table S6:** Results of PERMANOVA analysis testing the effect of various experimental factors on microbial diversity and differences between the groups in flow column experiment. Df - degree of freedom, SumOfSqs - sum of squares, R2 - coefficient of determination.

| Factor | Df | Sum of Squares | R² | F-value | p-value | Significant (p < 0.05) |
| --- | --- | --- | --- | --- | --- | --- |
| Medium type (NRM x R2A) | 1 | 1.1820 | 0.30484 | 19.2746 | 0.001 | Yes |
| Dynamic x static conditions | 1 | 1.2688 | 0.32721 | 20.6891 | 0.001 | Yes |
| Sample type (medium x swab) | 1 | 0.2002 | 0.05163 | 3.2643 | 0.011 | Yes |
| Residual | 20 | 1.2265 | 0.31632 |  |  |  |
| Total | 23 | 3.8775 | 1.00000 |  |  |  |
