# Supplementary material for: Microbiologically influenced corrosion (MIC) potential of bentonite microorganisms: implications for a deep geological repository for nuclear waste
Source: World J Microbiol Biotechnol. 2026 Jun 1;42(6):321. doi: 10.1007/s11274-026-05039-0 (PMC13226343; doi:10.1007/s11274-026-05039-0)
Supplement: Supplementary file 2 — Supplementary Material 2 (DOCX 3.92 MB) [file 11274_2026_5039_MOESM2_ESM.docx]

**Microbiologically influenced corrosion (MIC) potential of bentonite microorganisms: implications for a deep geological repository for nuclear waste**

**Supplementary Material 1**

Kateřina Černá^1*^, Saqlain Saqib Mukhtar^2^, Richard Bureš^2^, Gabriela Alfaro-Espinoza^3^, Andrea Koerdt^3^, Jakub Říha^1^, Veronika Hlavackova^1^, Jan Stoulil^2^

1 Technical University of Liberec, Institute for Nanomaterials, Advanced Technologies and Innovation, Studenstká 1402/2, 46117 Liberec, Czech Republic

2 University of Chemistry and Technology, Technická 5, 166 28 Prague, Czech Republic

3 Bundesanstalt für Materialforschung und -prüfung (BAM), Unter den Eichen 87, 12205 Berlin, Germany

*Corresponding author: [katerina.cerna1@tul.cz](mailto:katerina.cerna1@tul.cz), ORCID ID: 0000-0003-3351-6372

.


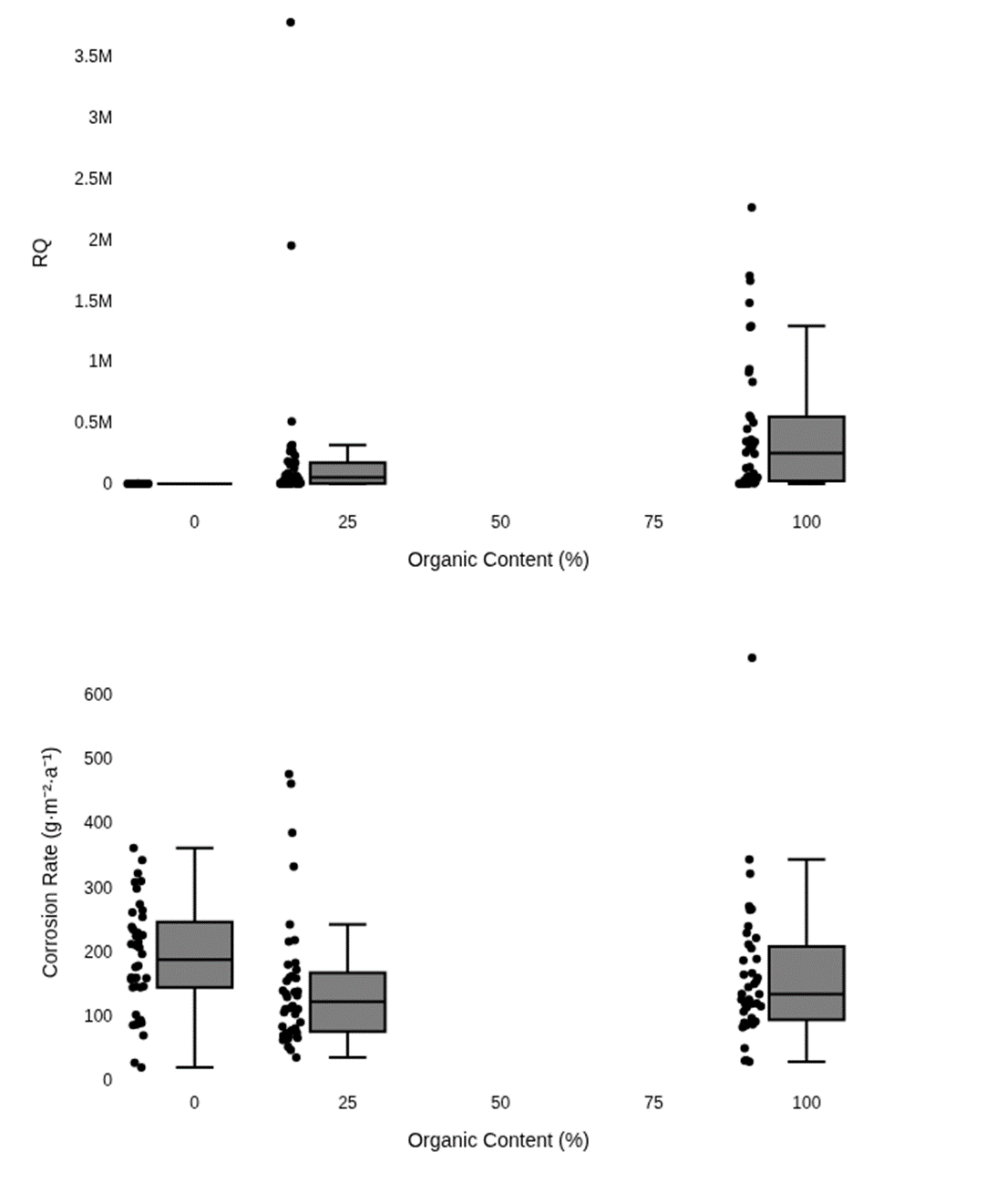


*Supplementary Figure S1: Relative microbial abundance (RQ) based on 16S rRNA gene copy numbers, M = × 10^6^ (up) and Corrosion rates (g·m^-2^·a^-1^)observed in biotic samples of different organic content (bottom).*


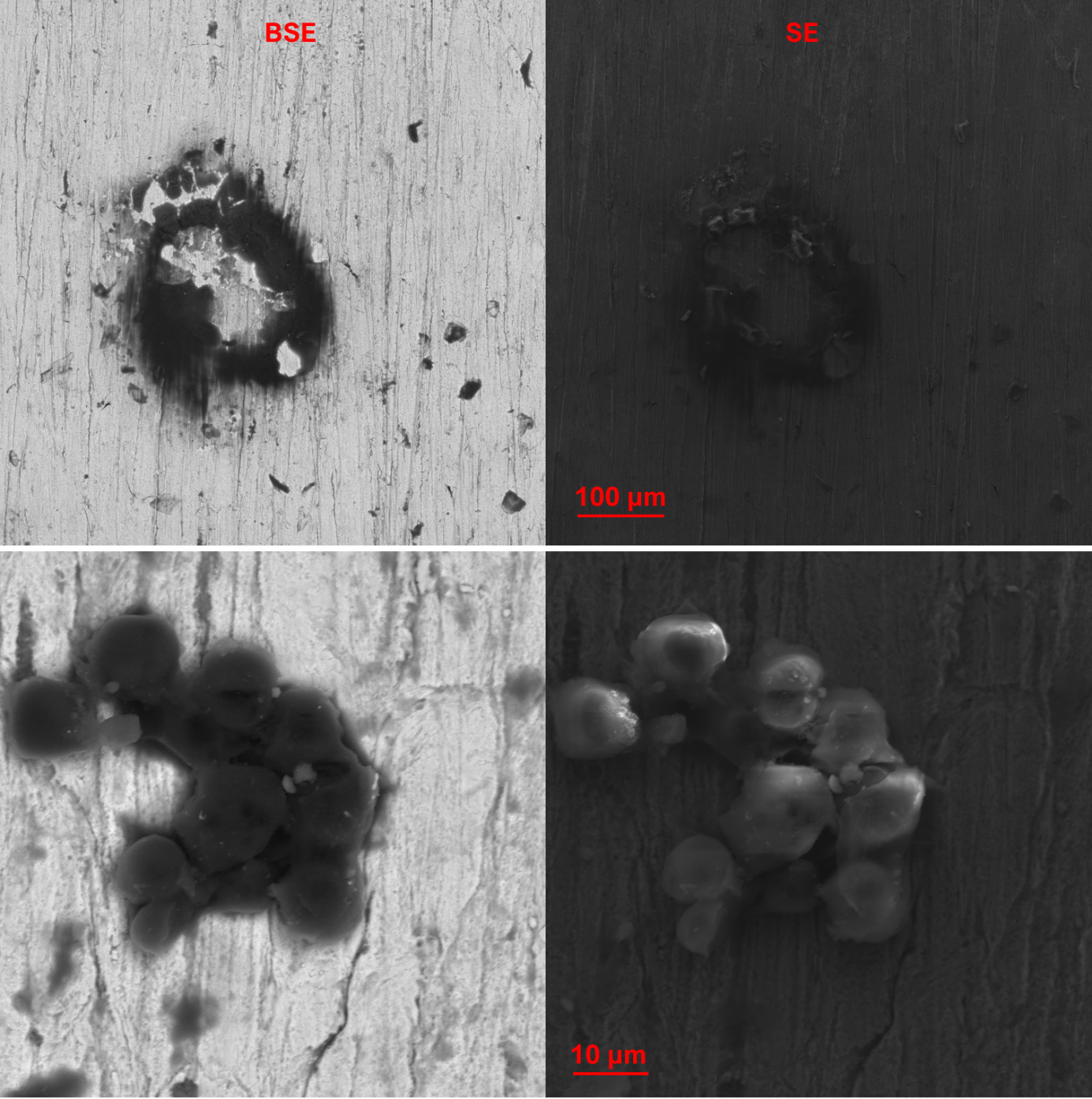


*Supplementary Figure S2: Backscattered electron (BSE) and secondary electron (SE) image of corrosion pit filled with a mixed layer of corrosion products and biofilm. Regions enriched in light elements are darker. Upper image – whole pit; lower image – detail of local biofilm and corrosion products.*

*
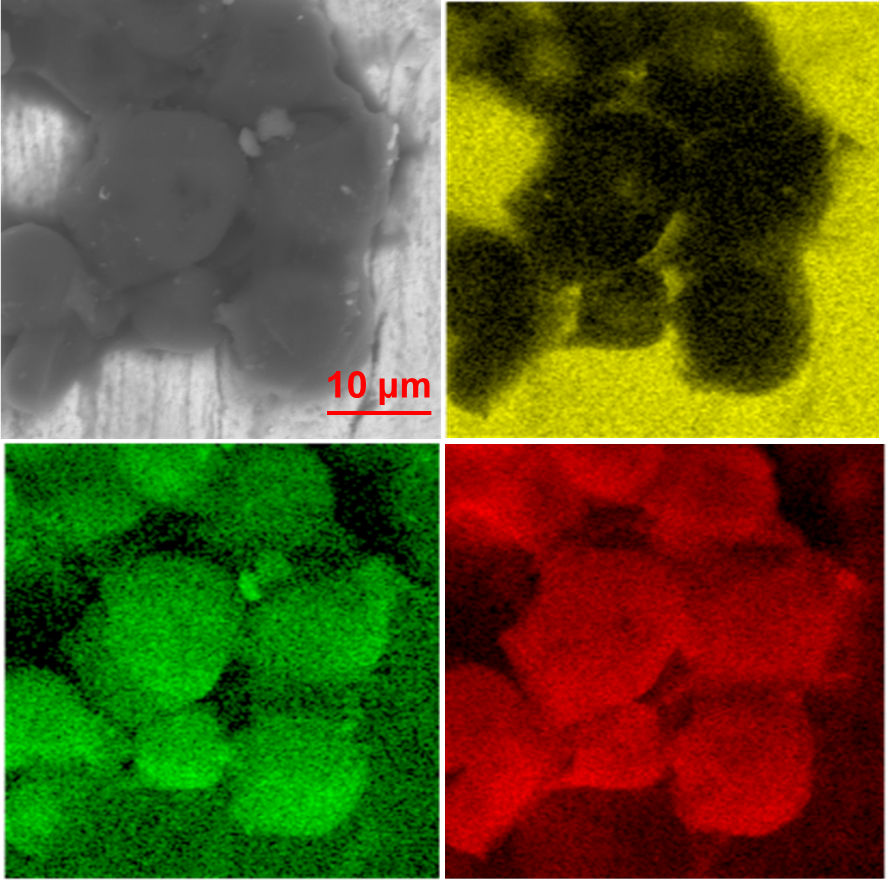
*

**C**

**O**

**Fe**

*Supplementary Figure S3: EDS maps of the biofilm/corrosion products layer.*

*
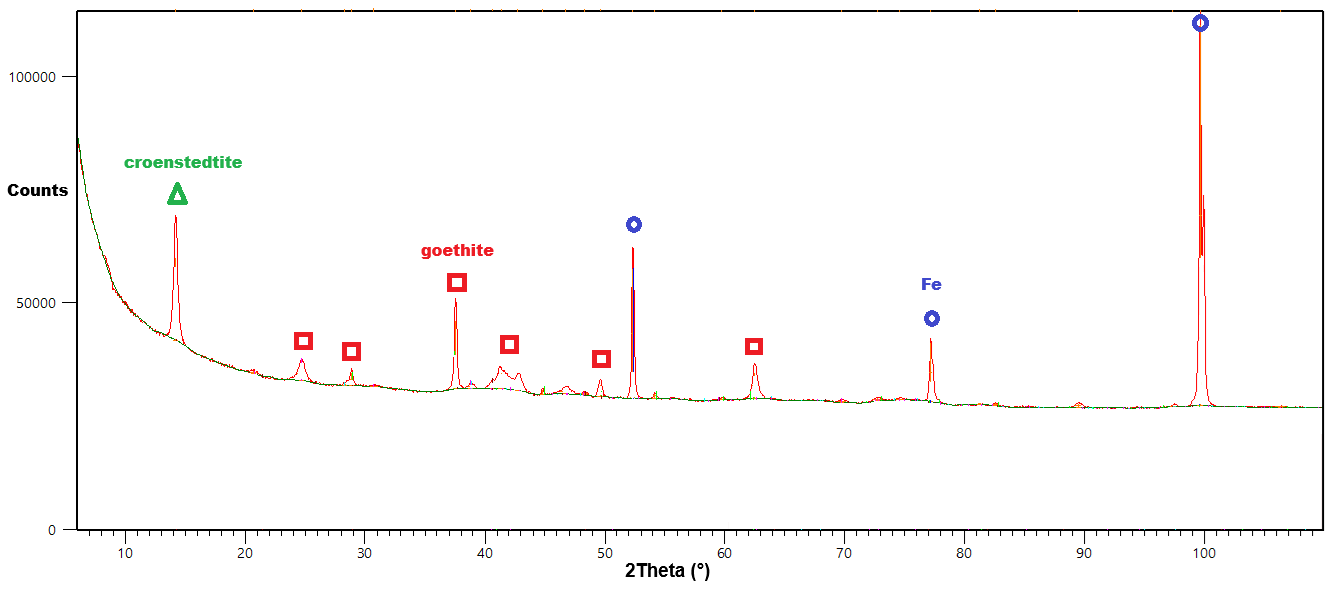
*

*Supplementary Figure S4: X-Ray Diffraction pattern of the sample exposed in flowing NRM*


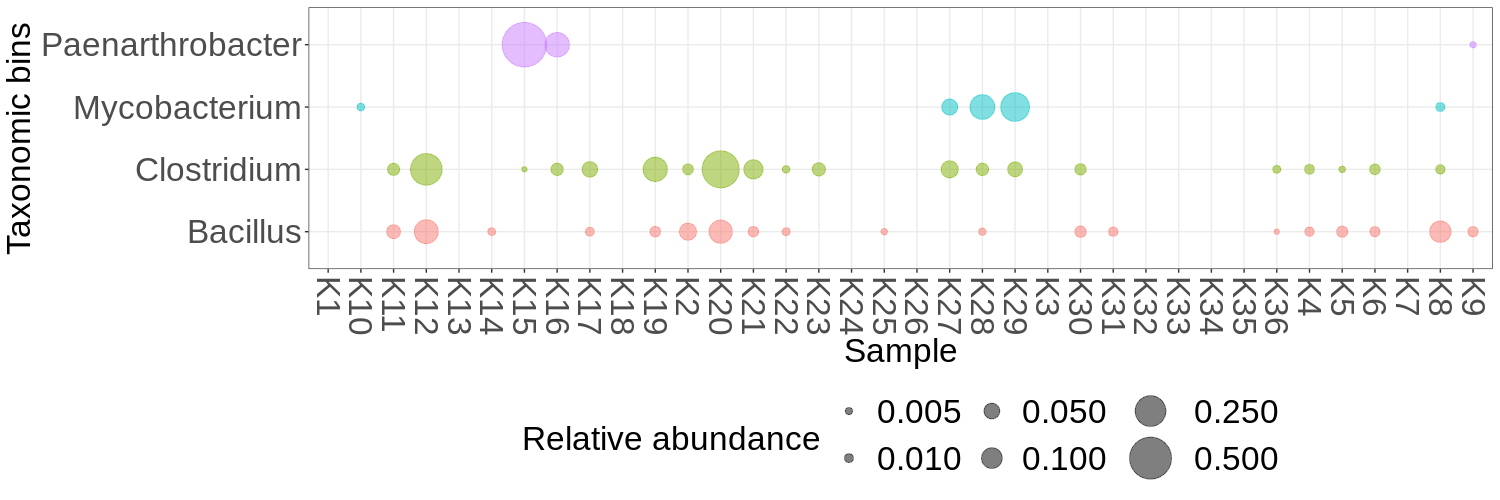


*Supplementary Figure S5: Microbial composition of the kit controls after Decontam treatment.*


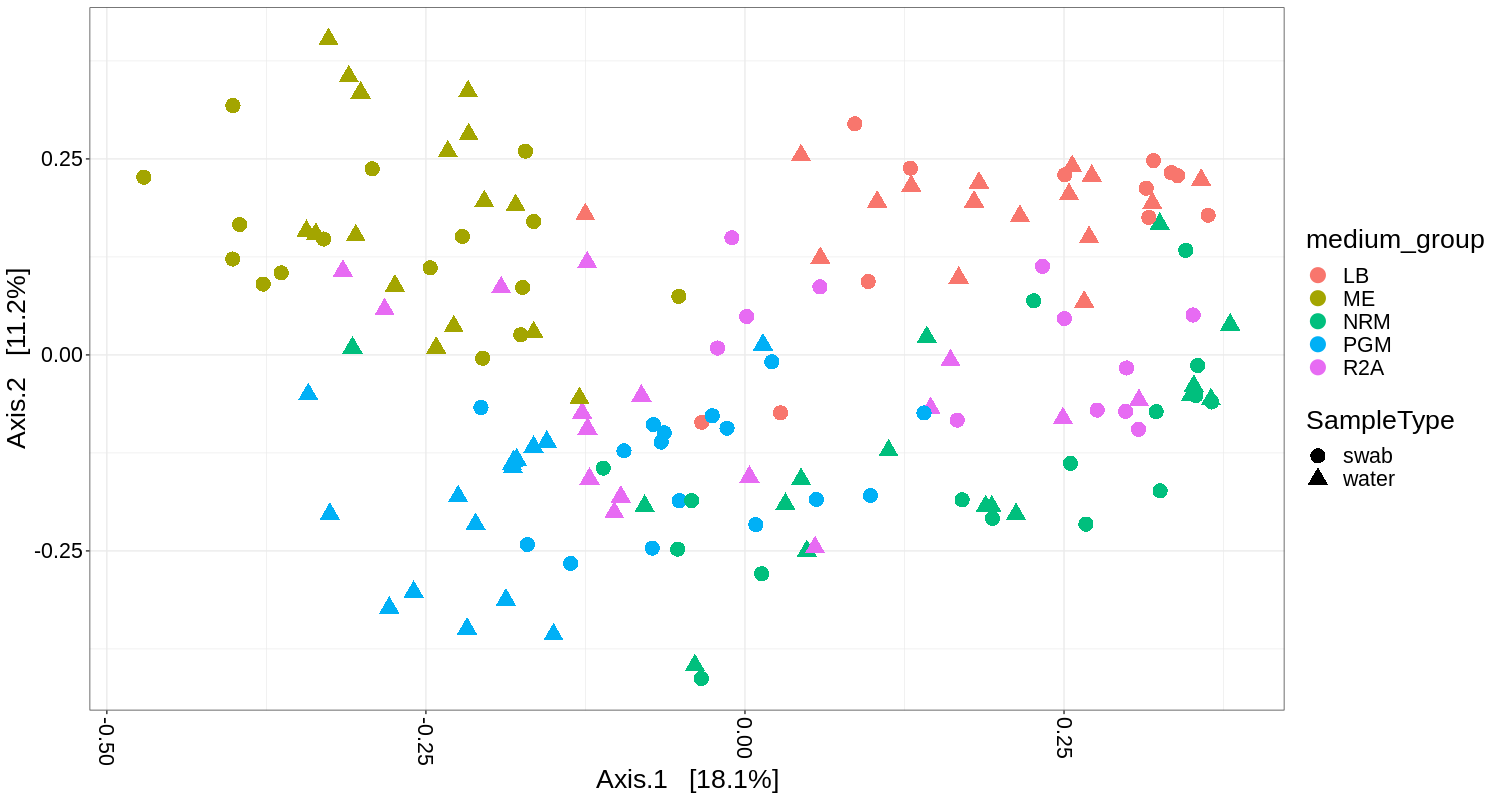


Supplementary Figure S6: PcoA analysis showing differences in microbial composition between culture samples. KC - kit control, PW - synthetic bentonite pore water including samples amended with nitrate, PGM - Postgate medium, LB - Luria Broth, NRM - Nitrate Broth, R2A - R2A medium, ME - medium 120 (Koblitz et al., 2023). 100/25/0 - 100 %/25 %/0 % of organic content compared to the original prescription.


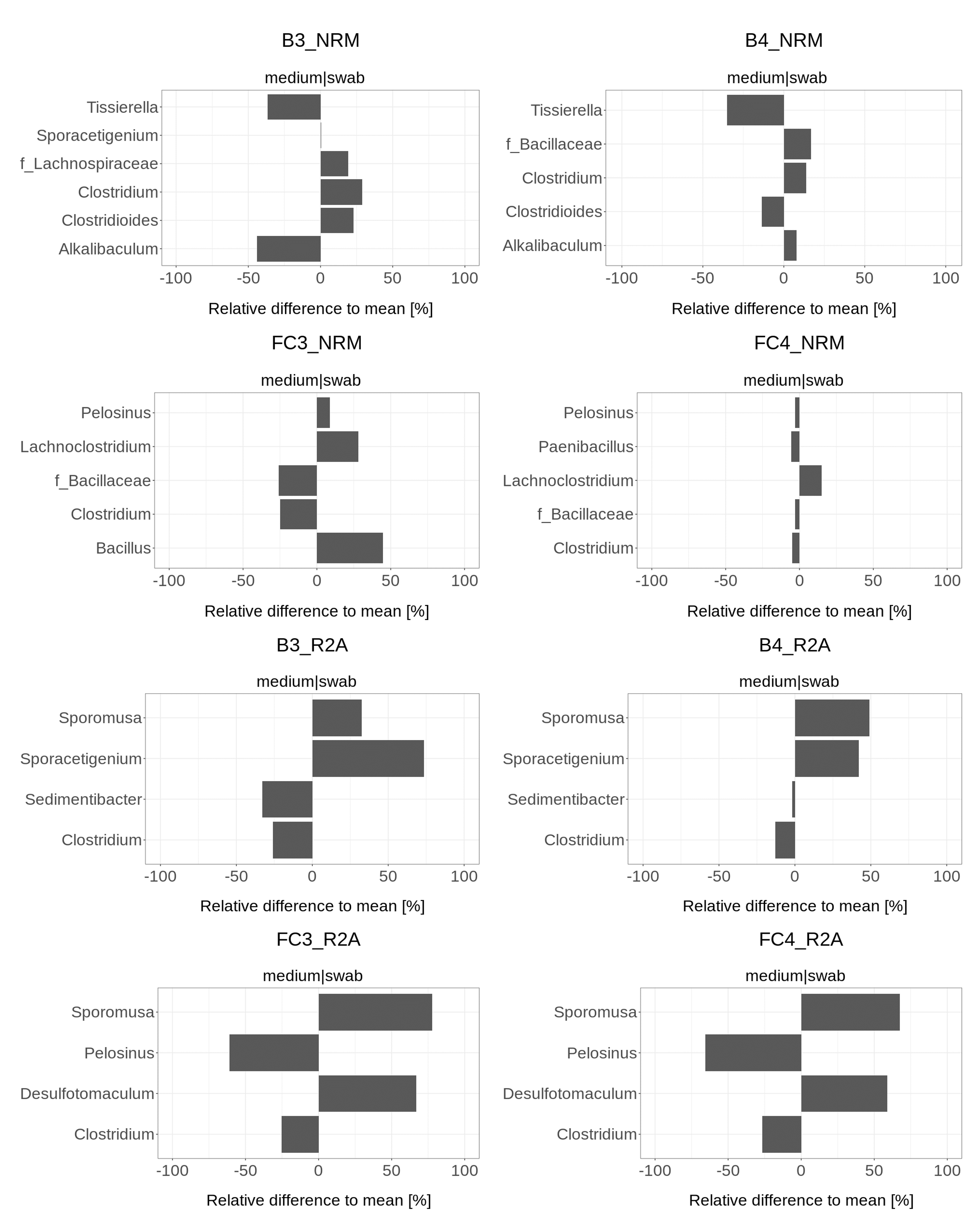


Supplementary Figure S7: Relative differences (%) from the mean abundances of dominant bacterial genera (≥5% mean relative frequency) are shown for each pair of bulk medium and the corresponding swab sample in inoculated dynamic (FC) and static (B) flow column samples. NRM/R2A - Nitrate Broth/R2A medium with 25% of organic content compared to the original prescription.
